# Inter-institutional variability in CT-to-mass-density conversion tables for helical tomotherapy: a national survey in Japan

**DOI:** 10.1093/jrr/rraf063

**Published:** 2025-10-16

**Authors:** Shogo Tsunemine, Shuichi Ozawa, Ryosei Nakada, Yasuo Shiota, Satoshi Kito, Hidetoshi Shimizu, Takashi Hashido, Takehiro Shiinoki, Yuto Kitagawa, Hiroshi Fukuma, Kentarou Sugi, Iori Sumida, Masumi Numano, Hideyuki Harada

**Affiliations:** Department of Radiation and Proton Office, Shizuoka Cancer Center, 1007 Shimonagakubo, Nagaizumi-cho, Sunto-gun, Shizuoka 411-8777, Japan; Hiroshima High-Precision Radiotherapy Cancer Center, 3-2-2 Futabanosato, Higashi-ku, Hiroshima 732-0057, Japan; Department of Radiation and Proton Office, Shizuoka Cancer Center, 1007 Shimonagakubo, Nagaizumi-cho, Sunto-gun, Shizuoka 411-8777, Japan; Division of Medical Physics, Iwata City Hospital, 512-3 Okubo, Iwata, Shizuoka 438-0002, Japan; Department of Radiation Oncology, Department of Radiology, Metropolitan Cancer and Infectious Disease Center Komagome Hospital, Tokyo, 3-18-22 Honkomagome, Bunkyo-ku, Tokyo 113-8677, Japan; Division of Medical Physics, School of Medical Sciences, Fujita Health University, 1-98 Dengakugakubo, Kutsukake-cho, Toyoake, Aichi 470-1192, Japan; Department of Medical Physics and Engineering, Division of Health Sciences, The University of Osaka Graduate School of Medicine, 2-15 Yamadaoka, Suita, Osaka 565-0871, Japan; Department of Radiation Oncology, Yamaguchi University Graduate School of Medicine, 1-1-1 Minami-Kogushi, Ube, Yamaguchi 755-0046, Japan; Department of Radiology, Nagoya City University Hospital, 1 Kawasumi, Mizuho-cho, Mizuho-ku, Nagoya 467-8602, Japan; Department of Radiology, Nagoya City University Hospital, 1 Kawasumi, Mizuho-cho, Mizuho-ku, Nagoya 467-8602, Japan; Physics and Clinical Support, Accuray, 2-2-1 Otemachi, Chiyoda-ku, Tokyo 100-0004, Japan; Department of Medical Physics and Engineering, Division of Health Sciences, The University of Osaka Graduate School of Medicine, 2-15 Yamadaoka, Suita, Osaka 565-0871, Japan; Physics and Clinical Support, Accuray, 2-2-1 Otemachi, Chiyoda-ku, Tokyo 100-0004, Japan; Department of Radiation and Proton Office, Shizuoka Cancer Center, 1007 Shimonagakubo, Nagaizumi-cho, Sunto-gun, Shizuoka 411-8777, Japan; Department of Radiation and Proton Center, Shizuoka Cancer Center, 1007 Shimonagakubo, Nagaizumi-cho, Sunto-gun, Shizuoka 411-8777, Japan

**Keywords:** CT–MD tables, CT modality, adaptive radiotherapy, half-phantom calibration, helical tomotherapy

## Abstract

This study evaluates current practices and challenges associated with computed tomography number-to-mass density (CT–MD) conversion tables in helical tomotherapy across Japan and explores directions for standardization and quality improvement amid the increasing adoption of adaptive radiotherapy (ART). A nationwide web-based survey was conducted across 34 institutions utilizing the Radixact system. Data were collected on CT acquisition protocols, calibration phantoms, density plugs, reconstruction algorithms, table registration timing and quality assurance (QA) frequency. Registered CT–MD tables were categorized by CT modality: Simulation CT (SimCT), ClearRT and CTrue. ClearRT tables were analyzed by phantom setup (full vs half), and CTrue tables by reconstruction method [filtered back projection (FPB) vs iterative reconstruction (IR)]. Inter-institutional variations in CT numbers and the number of data points were assessed. SimCT tables exhibited the widest variation in the number of data points (median = 10) and high-density CT numbers. ClearRT tables (median = 8) showed variations of up to 300 Hounsfield units (HU) in cortical bone; the half-phantom setup reduced inter-institutional variability. CTrue tables (median = 8) demonstrated high consistency, with negligible differences between IR and FPB. All plug CT numbers of CTrue remained within the tolerance defined by the American Association of Physicists in Medicine Task Group 148. However, CT numbers for air plugs varied by ~±30 HU, indicating inconsistent handling of air reference values. Additionally, 43% of institutions did not perform routine QA. Standardizing phantom geometry, air CT number handling and QA protocols—particularly using half-phantom calibration—may improve CT–MD table consistency and dose accuracy in ART.

## INTRODUCTION

On-board computed tomography (CT) systems are essential components of modern radiation therapy, enabling both image-guided radiation therapy and adaptive radiotherapy (ART). In ART, on-board CT imaging is performed immediately before each treatment session to capture anatomical changes, such as tumor shrinkage or patient weight loss. These updated images are used to recalculate dose distributions, and treatment plans may be re-optimized accordingly. This adaptive approach enhances dose accuracy, improves target conformity and reduces exposure to normal tissues, thereby contributing to safer and more precise treatments [1–3].

Among these systems, the Radixact platform (Accuray Inc., Sunnyvale, CA, USA)—the successor to tomotherapy—supports both helical and direct intensity-modulated radiation therapy delivery [4]. It incorporates two integrated imaging modalities: ClearRT, which uses kilovoltage CT (kVCT), and megavoltage CT (MVCT), referred to as CTrue. These modalities enable daily dose recalculation for ART [5, 6]. ClearRT provides higher-resolution images than CTrue, improving visualization of both bone and soft tissue, and potentially enhancing image registration and dose accuracy [7–9]. In contrast, CTrue achieves dose recalculation accuracy within 2%, despite its lower image quality [10–12]. Early clinical series have demonstrated the feasibility of Radixact-based adaptive workflows with ClearRT or CTrue [8, 13]. However, according to expert opinions and current clinical practice trends, widespread routine implementation remains limited.

The accuracy of heterogeneity correction in dose calculations depends critically on the quality assurance (QA) of the CT-to-mass density (CT–MD) conversion table, which defines the relationship between CT numbers and MD [14, 15]. Errors in this conversion can lead to inaccurate estimations of radiological path length, necessitating high precision. However, the construction of CT–MD tables vary significantly across institutions, including differences in phantom setup, imaging protocols and calibration methods. Even within a single system like Radixact, variations in imaging energy and image characteristics between ClearRT and CTrue can lead to discrepancies in dose calculations. Previous studies have shown that inconsistencies in CTrue-based CT–MD tables can negatively affect dose accuracy [16]. Therefore, establishing modality-specific CT–MD tables and conducting regular QA are essential for accurate ART implementation [6, 17].

Despite this need, few studies have conducted comprehensive multi-institutional comparisons of CT–MD table construction. Research focusing specifically on CT–MD tables generated using integrated on-board imaging systems remains limited [18, 19]. In particular, no systematic investigation has assessed inter-institutional variability in CT–MD tables for both ClearRT and CTrue on the Radixact platform.

The objective of this study is to characterize current institutional practices for CT–MD table construction using the Radixact Radiation treatment planning system (RTPS) in Japan. We examined the number of calibration points, imaging protocols and variations in CT numbers under different acquisition conditions, as these factors directly affect CT–MD table accuracy. This study aims to support standardization efforts and improve the reproducibility and QA of high-precision ART.

## METHODS

### Study population and data collection

This study conducted a web-based survey targeting Japanese institutions equipped with Radixact systems incorporating Simulation CT (SimCT), ClearRT and CTrue. The objective was to investigate the methodologies used to construct CT–MD tables for each imaging modality, as registered in the Precision RTPS (Accuray Inc., Sunnyvale, CA, USA). Data were collected via an online questionnaire (RTQM Inc., Hiroshima, Japan) administered between September and December 2024. To enable inter-modality comparisons of CT–MD table precision, the survey included questions on general practices (e.g. phantom setup, calibration methods) as well as CT numbers and corresponding MD values for each material.

A total of 34 institutions responded regarding SimCT and CTrue, and 29 institutions provided data for ClearRT. Detailed responses were collected for each modality. The questionnaire was distributed to 41 institutions, with valid responses obtained from all 34 Radixact-equipped centers; the remaining seven institutions did not respond.

### Multi-center questionnaire survey

#### SimCT

For SimCT, the survey collected information on the CT scanner manufacturer, scan parameters (including tube voltage, field of view (FOV) and slice thickness), acquisition mode (helical vs non-helical), the software used for CT number extraction, and the calibration phantom employed for constructing the CT–MD table. Additionally, the CT–MD tables registered in the RTPS were analyzed to evaluate inter-institutional variability.

Furthermore, we compared the variability observed in the complete SimCT dataset with that in a subgroup dataset in which the FOV and phantom type were fixed.

#### ClearRT (kVCT)

**Fig. 1:**
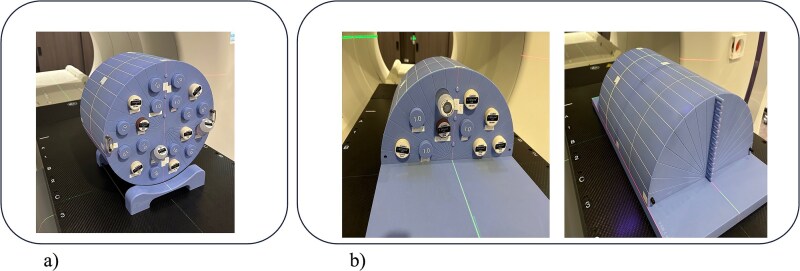
Phantom configurations used for CT–MD table calibration. (a) Full-phantom method: all tissue-equivalent plugs are inserted into the cylindrical phantom simultaneously to simulate a uniform surrounding medium. (b) Half-phantom method: Density plugs are embedded in a semi-cylindrical phantom and covered with additional phantom material to eliminate air interfaces and minimize beam-hardening effects.

For the ClearRT system, the survey collected information on the calibration phantom used for constructing the CT–MD table, the manufacturer of the density plugs, the frequency of QA procedures specifically related to the CT–MD table, and the date of CT–MD table registration. Additionally, respondents were asked to specify which of the following two measurement methods was used to generate the CT–MD table:

Full-phantom method: all tissue-equivalent density plugs were inserted into the phantom with a slight protrusion from the surface, allowing CT acquisition and subsequent CT number measurements for each plug (Fig. 1a).

Half-phantom method: the tomo phantom was divided into upper and lower halves. The plugs were inserted into the upper half and fully embedded by covering them with the lower half. This configuration minimizes external influences on CT numbers, such as beam hardening and air effects (Fig. 1b).

In the ClearRT system, up to 10 CT–MD tables can be registered based on the clinical site and scanning protocol. Of these, data corresponding to the ‘Thorax Normal (FOV 440 mm)’ protocol—commonly used for CT number calibration—are presented in the main text, while details for the remaining tables are provided in supplementary data Fig. A. The tube voltage (kV) in the ClearRT system was fixed according to the anatomical region being scanned, based on manufacturer-defined protocols: 100 kV for the head, 120 kV for the thorax and whole-body and 140 kV for the pelvis. Therefore, the kV setting was consistent across institutions for each scan type. Given the significant variation expected between tables due to phantom configuration (full vs half), inter-institutional comparisons were stratified accordingly.

#### CTrue (MVCT)

For CTrue, the survey collected information on the calibration phantom, the manufacturer of the tissue-equivalent density plugs, the date of CT–MD table registration, the frequency of QA procedures and the reconstruction algorithm used. CTrue supports multiple reconstruction methods, including filtered back projection (FBP), iterative reconstruction (IR) and IR optimized for soft tissues. As each institution independently selects the reconstruction algorithm, variations in the CT–MD tables may arise depending on the chosen method. This study evaluated differences in CT–MD tables generated using the three reconstruction algorithms. For comparative analysis, institutions were grouped based on whether they employed FBP- or IR-based reconstruction.

### Analytical methods for the number of data points in CT-to-MD conversion tables

For each CT modality used in the clinical practice (SimCT, ClearRT and CTrue), the number of data points included in the CT–MD tables was collected from each institution. Furthermore, the number of data points used at each institution was compared with the recommended values (typically eight materials) described in widely cited white papers for each modality to evaluate the discrepancies between actual clinical practice and established guidelines [20].

## RESULTS

### SimCT

Table 1 summarizes responses from 34 domestic institutions regarding the construction of CT–MD tables for SimCT. Canon Medical scanners were the most commonly used (57%), followed by SIEMENS (23%) and GE Healthcare (20%). A tube voltage of 120 kV was predominantly selected (91%). The most frequently used reconstructed slice thickness was 2 mm (60%), followed by 2.5 mm (14%). A reconstruction FOV of 500 mm was employed by the majority of institutions (59%), and helical acquisition was adopted at 91% of sites.

**Table 1:** Summary of institutional practices for CT–MD table creation across SimCT, ClearRT and CTrue modalities

Category	Modality	Most common (1st)	2nd most common	3rd most common	Others unknown (%)
Scanner manufacturer	SimCT	Canon (57%)	SIEMENS (23%)	GE healthcare (20%)	
Tube voltage	SimCT	120 kVp (91%)	100 kVp (6%)		3%
Slice thickness	SimCT	2.0 mm (60%)	2.5 mm (14%)	1.0 mm (8%)	18%
Scan FOV	SimCT	500 mm (59%)	550 mm (14%)	700 mm (9%)	18%
Scan mode	SimCT	Helical (91%)	Non-helical (6%)		3%
Calibration phantom	SimCT	SN-SW-Tomo (43%)	Gammex 467 phantom (29%)	CIRS/GAMMEX (11%)	6%
	ClearRT	SN-SW-Tomo (52%)	CIRS Advanced (28%)	GAMMEX / VW phantom (10%)	10%
	CTrue	SN-SW-Tomo (70%)	Gammex 467 phantom (9%)	VW phantom / CIRS (9%)	12%
Density plug type	ClearRT	TomoTherapy-specific (83%)	CIRS (10%)	Gammex (4%)	3%
	CTrue	TomoTherapy-specific (94%)	CIRS (3%)	Gammex (3%)	
Table registration timing	ClearRT	During commissioning (62%)	After clinical start (38%)		
	CTrue	During commissioning (85%)	After clinical start (15%)		
QA frequency	ClearRT	Not done (50%)	Annually (29%)	Monthly (18%)	3%
	CTrue	Not done (43%)	Monthly/annually (24%)	Semiannually (9%)	
CT number Acquisition method	ClearRT	Full phantom (55%)	Half phantom (28%)	By protocol (17%)	
Reconstruction algorithm	CTrue	FBP (58%)	IR general (21%)	IR soft tissue (9%)	12%

Each row represents a specific procedure or parameter relevant to the construction of the CT–MD table. For each imaging modality, the most frequently adopted methods are listed in descending order of prevalence, while less common responses are categorized as ‘Others’ where appropriate. Abbreviations: SN-SW-Fast, Sun Nuclear solid water tomo phantom; VW phantom, virtual tomo phantom.

The most commonly used calibration phantom was the sun nuclear solid water tomo phantom (Sun Nuclear Corporation, Melbourne, FL, USA) (43%), followed by the Gammex 467 phantom (29%), and the CIRS Advanced Electron Density phantom (Med-Cal, Middleton, WI, USA) and Gammex (Sun Nuclear Corporation, Melbourne, FL, USA) (11% each).

**Fig. 2:**
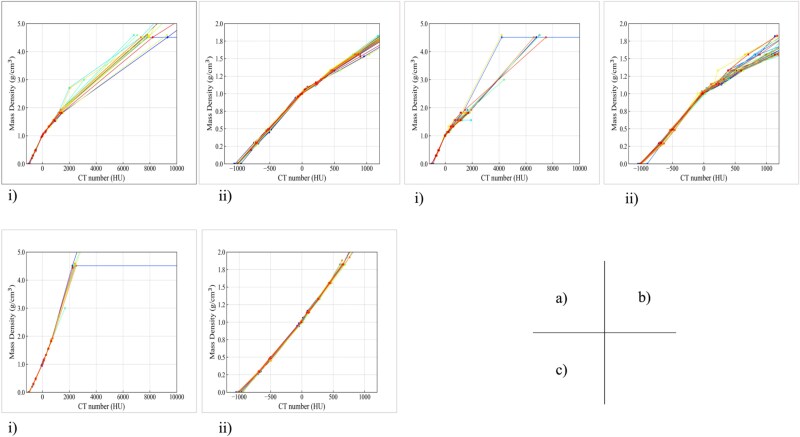
CT–MD calibration curves obtained from multiple institutions using (a) SimCT, (b) ClearRT and (c) CT_RUE_. Each modality includes two subpanels: (i) full MD range (0–5.0 g/cm^3^) and (ii) low-to-intermediate range (air to bone, 0–2.0 g/cm^3^). Colored lines represent individual institutional CT–MD tables, illustrating inter-institutional variation across different density regions.

Figure 2a shows the CT–MD tables registered in the RTPS across all participating institutions. Across the full CT number range [−1000 to 10 000 Hounsfield units (HU)], substantial inter-institutional variability was observed, particularly in the high-density region (>1000 HU). In contrast, the low-density range (−1000 to 1000 HU) exhibited more consistent trends among institutions, though minor differences in slope and CT–MD conversion were still apparent. The air–lung equivalent region (~−1200 to −500 HU) showed good consistency, while the soft tissue range (−500 to 400 HU) exhibited tight clustering ~0 HU. In contrast, the bone region (400 to 1200 HU) demonstrated notable variation, with divergence increasing at higher densities. A graphical summary is provided in supplementary data Fig. B.

Table 2 presents a statistical summary of CT numbers and mass densities associated with each tissue-equivalent plug. Minimal variation was observed for reference materials: the mean CT number for water was 1.4 ± 6.3 HU, and for air, −995.9 ± 12.9 HU. In contrast, greater variability was found for higher-density plugs. Lung-equivalent plugs LN-300 and LN-450 yielded mean CT numbers of −703.6 ± 14.4 HU and − 518.8 ± 17.5 HU, respectively. Bone-equivalent plugs showed increasing variability with density: CB-30% (468.3 ± 28.1 HU), CB-50% (848.0 ± 48.8 HU), and cortical bone (1284.5 ± 78.5 HU). Notably, cortical bone exhibited the largest standard deviation (SD), indicating substantial inter-institutional variability in this region.

**Table 2:** Summary statistics for eight tissue-equivalent density plugs used in SimCT CT–MD tables collected from 34 institutions. Colors indicate individual institutions. Axis ranges differ between panels to optimize visualization of relevant CT number and density ranges for each dataset

		MD (g/cm^3^)	CT number (HU)
Tissue-equivalent density plug	*n* ^*^	Mean (min–max)	Mean ± 1SD
Air	34	0.001 (0.001–0.01)	−995.9 ± 12.9
LN-300 lung	32	0.29 (0.26–0.30)	−703.6 ± 14.4
LN-450 lung	34	0.48 (0.45–0.50)	−518.8 ± 17.5
True water	34	1.00 (1.00–1.00)	1.4 ± 6.3
Inner bone	32	1.14 (1.11–1.16)	219.9 ± 11.2
CB-30%	31	1.33 (1.33–1.34)	468.3 ± 28.1
CB-50%	34	1.56 (1.53–1.56)	848.0 ± 48.8
Cortical bone	32	1.82 (1.82–1.83)	1284.5 ± 78.5

For each plug, mass-density (MD) is reported as the mean with range (min–max), and CT numbers are reported as the mean ± 1 SD. n^*^ denotes the number of facilities that contributed valid datasets for the corresponding plug.

To evaluate the impact of acquisition variability, a subgroup analysis was conducted using only data acquired with the most common scan parameters: a 500 mm scan FOV and the Sun Nuclear SN-SW-Tomo phantom (*n* = 8–9 per plug). The results are summarized in Supplementary Table S1 Comparison of CT–MD Calibration Data for the Overall Data Set and the Matched-Conditions Subgroup, SDs in low-density materials were slightly reduced (e.g. true water: 6.3 → 4.8 HU; inner bone: 11.2 → 9.8 HU). In contrast, high-density plugs showed minimal improvement, with SDs remaining large (e.g. CB-50%: 48.8 → 42.3 HU; cortical bone: 78.5 → 78.2 HU).

### ClearRT (kVCT)

Table 1 also summarizes nationwide practices related to CT–MD table calibration and QA for ClearRT. The most commonly used calibration phantom was the sun nuclear solid water daily phantom (52%), followed by the CIRS advanced electron density phantom (28%), and the virtual water and Gammex 467 phantoms (10% each). Regarding density plugs (83%) of institutions used tomotherapy-specific Gammex plugs, while CIRS (10%), Gammex (4%) plugs were used less frequently.

CT–MD tables were most often registered during system commissioning (62%), with the remaining 38% registered afterward. QA frequency varied: 50% of institutions did not perform routine QA, while 29% conducted annual checks, 18% monthly and 3% semi-annually. Measurement configurations also differed: 55% used the full-phantom method, 28% the half-phantom method and 17% selected the method based on imaging protocol.

Figure 2b shows the CT–MD tables acquired using the ‘Thorax FOV 440 mm, Normal’ protocol. Across the full CT number range (−1000 to 10 000 HU) [Fig. 2b(i)], most institutional curves overlapped below ~1000 HU but diverged considerably at higher values, with some tables assigning densities >4.0 g/cm^3^ to CT numbers between 3000 and 7000 HU. In the low-density range (−1000 to 1000 HU) [Fig. 2b(ii)], trends were mostly linear with modest variation in slope and intercept. In the air-equivalent region (−1200 to −500 HU), values were tightly clustered, although 2–3 institutions deviated by 30–40 HU, suggesting minor calibration inconsistencies. In contrast, the bone region (400 to 1200 HU) exhibited large discrepancies: variation exceeded 150 HU at ~1.8 g/cm^3^ and 250 HU beyond 2.0 g/cm^3^, indicating substantial inter-institutional differences in high-density calibration despite protocol standardization. Visualizations are provided in supplementary data Fig. A6, which also includes the CT–MD table results for additional imaging protocols.

Table 3 presents a statistical summary of CT numbers and mass densities for nine tissue-equivalent plugs scanned using the same ClearRT protocol. Results are presented for all participating institutions (*n* = 26–29; the exact number differs by density plug because some sites did not submit measurements for every material). Data were also analyzed according to phantom-setup method: the full-phantom configuration contributed 13–19 datasets and the half-phantom configuration 8–9 datasets, depending on the plug examined. Air and water showed the least variation (−1004 ± 11 HU and − 21 ± 24 HU, respectively). Lung-equivalent plugs (LN-300 and LN-450) had means of −680 HU and − 500 HU, with SDs around 20 HU. High-density materials showed greater variability: SDs were 75 HU (inner bone), 99 HU (CB-30%), 188 HU (CB-50%) and >250 HU (cortical bone).

**Table 3:** Comparison of CT number and MD for eight tissue-equivalent density plugs used in CT–MD tables registered in precision, constructed from ClearRT Thorax FOV 440 mm (normal) images using full-phantom and half-phantom methods, as well as the combined dataset

		Entire data			Full phantom method			Half phantom method	
		MD (g/cm^3^)	CT number (HU)		MD (g/cm^3^)	CT number (HU)		MD (g/cm^3^)	CT number (HU)
Tissue-equivalent density plug	*n* ^*^	Mean (min–max)	Mean ± 1SD	n^*^	Mean (min–max)	Mean ± 1SD	n^*^	Mean (min–max)	Mean ± 1SD
	26	0.001 (0.001–0.001)	−1004.3 ± 11.0	17	0.001 (0.001–0.001)	−1005.0 ± 13.5	8	0.001 (0.001–0.001)	−1002.7 ± 2.8
LN-300 lung	29	0.29 (0.28–0.31)	−679.9 ± 20.4	19	0.29 (0.28–0.31)	−673.5 ± 22.1	9	0.29 (0.29–0.30)	−692.4 ± 8.3
LN-450 lung	29	0.48 (0.45–0.50)	−500.4 ± 21.3	19	0.48 (0.45–0.50)	−500.2 ± 22.9	9	0.49 (0.47–0.49)	−500.0 ± 20.1
True water	29	1.00 (1.00–1.02)	−21.4 ± 24.1	19	1.00 (1.00–1.02)	−23.9 ± 25.8	9	1.00 (1.00–1.00)	−17.0 ± 22.0
Inner bone	29	1.16 (1.13–1.22)	238.4 ± 75.2	19	1.16 (1.13–1.22)	232.1 ± 88.2	9	1.15 (1.13–1.21)	251.3 ± 45.0
CB-30%	29	1.33 (1.33–1.34)	508.8 ± 98.6	19	1.33 (1.33–1.34)	478.2 ± 98.2	9	1.33 (1.33–1.34)	563.4 ± 76.1
CB-50%	29	1.56 (1.56–1.56)	990.0 ± 187.5	19	1.56 (1.56–1.56)	924.4 ± 193.3	9	1.56 (1.56–1.56)	1111.5 ± 96.4
Cortical bone (1.82 g/cm^3^)	22	1.82 (1.82–1.83)	1512.6 ± 257.0	13	1.82 (1.82–1.83)	1399.0 ± 227.5	8	1.82 (1.82–1.82)	1670.2 ± 224.3
Cortical bone (1.92 g/cm^3^)	7	1.92 (1.92–1.93)	1698.4 ± 334.4	6	1.92 (1.92–1.93)	1660.0 ± 348.9	1	1.92 (1.92–1.92)	1929.1^†^ —

For each tissue-equivalent plug, the MD is presented as the mean value along with its range (Min–Max), and CT numbers are presented as the mean ± one SD. The table includes data from three sources: full-phantom calibration, half-phantom calibration and a combined dataset encompassing all available data. ^*^n denotes the number of facilities contributing to valid datasets for each plug. ^†^Data from a single institution; SD not applicable.

Stratified analysis revealed minimal differences in low-density plug measurements; however, the half-phantom method consistently resulted in higher CT numbers for dense materials: +60 HU for CB-30%, +190 HU for CB-50% and + 270 HU for cortical bone (1.82 g/cm^3^). Furthermore, the SDs were smaller in the half-phantom group, indicating improved consistency in high-density calibration. Similar trends were observed across other protocols (supplementary data), highlighting the impact of phantom configuration on CT–MD table accuracy in high-density regions. Across the ten ClearRT protocols presented in Supplementary Fig. A, low-density materials (air, water, lung) showed tight clustering with SD ≤ 25 HU, whereas variability widened progressively with density: CB-30% SD ≈ 80 HU and cortical bone SD ≈ 200–290 HU. The comprehensive side-by-side comparisons in Supplementary Materials (Figs A1–A10) reveal that the half-phantom method consistently narrows the spread of high-density CT numbers by 20–35% relative to the full-phantom method, irrespective of scan FOV or anatomical preset, supporting its robustness across ClearRT protocols.

### CTrue MVCT

Table 1 also summarizes nationwide practices for CT–MD table calibration and QA in CTrue. The most frequently used calibration phantom was the Sun Nuclear solid water tomo phantom (70%), followed by the Gammex 467 phantom and the CIRS Advanced Electron Density phantom (both 9%).

Tomotherapy-specific Gammex plugs were used by 94% of institutions, with general-purpose Gammex and Sun Nuclear plugs used by 3% each.

CT–MD tables were typically registered during system commissioning (85%), while the remaining 15% were registered after clinical implementation. QA practices varied: 43% of institutions did not perform routine QA, while 24% conducted monthly checks, 24% annually and 9% semi-annually.

Regarding reconstruction methods, FBP was the most commonly used (58%), followed by general IR (21%) and IR for soft tissue (9%). In 12% of institutions, the reconstruction algorithm was not specified.

Figures 2c(i and ii) show the CT–MD tables constructed using CTrue across 34 institutions. Compared to SimCT and ClearRT, the CTrue tables showed lower inter-institutional variability. In the high-density region, CTrue curves were more tightly aligned across institutions. A graphical summary is provided in supplementary data Fig. 100.

CT number distributions for six density plugs (LN-300, LN-450, True water, CB-30%, CB-50% and cortical bone) were compared between FBP- and IR-based methods. No significant differences were observed in mean CT numbers or SDs between the groups. Table 4 summarizes inter-institutional means and SDs across different density regions. Although minor differences were noted between the IR and FBP groups, the variability was small and showed no systematic trend. CT numbers for all plugs remained within the American Association of Physicists in Medicine (AAPM) Task Group 148 tolerance limits (±50 HU for bone, ±30 HU for water, ±50 HU for lung) for intra-institutional acceptance and periodic QA. In this study, no individual center’s baseline exceeded its own acceptance band, indicating that all participating sites satisfied the TG-148 criteria used for their internal QA. Air plugs exhibited the largest variability, with an SD of approximately 30 HU, a metric not specified in TG-148 [21].

**Table 4:** Comparison of CT numbers and mass densities for tissue-equivalent plugs in CT–MD tables constructed from CTRUE images, categorized by reconstruction algorithm (FBP vs IR) and combined dataset

		Entire data			Full phantom method			Half phantom method	
		MD (g/cm^3^)	CT number (HU)		MD (g/cm^3^)	CT number (HU)		MD (g/cm^3^)	CT number (HU)
Tissue-equivalent density plug	*n* ^*^	Mean (min–max)	Mean ± 1SD	n^*^	Mean (min–max)	Mean ± 1SD	n^*^	Mean (min–max)	Mean ± 1SD
	31	0.001 (0.001–0.001)	−995.4 ± 27.2	22	0.001 (0.001–0.001)	−994.4 ± 27.9	8	0.001 (0.001–0.001)	−998.2 ± 28.8
LN-300 lung	32	0.29 (0.26–0.30)	−686.4 ± 9.9	22	0.29 (0.27–0.30)	−686.3 ± 9.6	9	0.29 (0.26–0.30)	−686.4 ± 11.7
LN-450 lung	33	0.48 (0.45–0.50)	−505.3 ± 7.6	22	0.48 (0.45–0.50)	−503.7 ± 8.0	10	0.48 (0.46–0.50)	−508.5 ± 6.3
True water	32	1.00 (1.00–1.02)	−6.2 ± 8.1	21	1.00 (1.00–1.02)	−7.0 ± 7.4	19	1.00 (1.00–1.00)	−4.7 ± 10.1
Inner bone	27	1.14 (1.13–1.16)	87.7 ± 11.8	17	1.14 (1.13–1.15)	88.9 ± 12.4	9	1.14 (1.13–1.16)	86.6 ± 10.8
CB-30%	32	1.33 (1.33–1.34)	261.2 ± 10.2	22	1.33 (1.33–1.34)	260.3 ± 11.1	9	1.33 (1.33–1.34)	263.5 ± 8.2
CB-50%	33	1.56 (1.53–1.56)	440.7 ± 12.5	22	1.56 (1.53–1.56)	441.9 ± 12.2	10	1.56 (1.53–1.56)	438.7 ± 13.9
Cortical bone (1.82 g/cm^3^)	31	1.82 (1.82–1.88)	650.4 ± 15.1	20	1.82 (1.82–1.88)	653.2 ± 14.5	10	1.82 (1.82–1.82)	645.0 ± 16.4

For each tissue-equivalent plug, the MD is presented as the mean value along with its range (min–max), and CT numbers are presented as the mean ± one SD. The table includes data from three sources: full-phantom calibration, half-phantom calibration and a combined dataset encompassing all available data. *n denotes the number of facilities contributing to valid datasets for each plug.

### Comparison of data points used in CT–MD table

The number of data points used in the CT–MD tables varied across the three CT modalities. For SimCT, both the mean and median were 10, with a range of 8–20, indicating moderate variability among institutions. ClearRT showed a mean of 9 and a median of 8, with a narrower range of 8–18, suggesting more consistent implementation. CTrue also had a mean and median of 9, but with a slightly broader range of 7–18. These results suggest that while most institutions adhered to the general recommendation of 8–10 calibration points, some—particularly those using SimCT—adopted more granular CT–MD tables.

## DISCUSSION

### SimCT

This study evaluated CT–MD tables derived from SimCT across 34 institutions in Japan and identified inter-institutional variability in calibration procedures. The survey revealed differences in calibration phantoms, reconstructed slice thickness, scan FOV and the software used for CT number extraction. Analysis of the CT–MD tables showed the greatest discrepancies in the high-density region (>1000 HU), likely due to beam hardening effects, variations in plug composition and scanner-specific x-ray spectra. These discrepancies may increase dose calculation uncertainty in anatomies containing dense structures, such as the head and neck, pelvis or spine. Further validation using phantom measurements and clinical data is needed to assess the clinical significance of these findings.

In contrast, strong agreement was observed near water and air, supporting the reliability of dose calculations in lung or gas-containing regions. The mean CT number for the water plug was 1.4 HU (SD: 6.3 HU), and for air was −996 HU (SD: 13 HU). Most institutions met or closely approached the AAPM TG-66 tolerance of ±5 HU for water and converged near −1000 HU for air, indicating that baseline calibration procedures were generally appropriate [22]. These subgroup results indicate that, although acquisition parameters such as scan FOV and phantom type influence CT number variability, substantial residual variation—particularly in high-density plugs—remains even under harmonized imaging conditions (Supplementary Table S1). The SD for true water decreased from 6.3 to 4.8 HU, and for inner bone from 11.2 to 9.8 HU, indicating modest improvement in low-density regions. In contrast, variability in high-density plugs showed little change: CB-50% decreased from 48.8 to 42.3 HU, and cortical bone from 78.5 to 78.2 HU. This suggests that residual variability likely arises from procedural factors (e.g. ROI placement, reconstruction settings, operator technique) and scanner-dependent characteristics (e.g. manufacturer-specific algorithms and hardware). Although these sources cannot be conclusively identified in a retrospective multi-institutional study, our findings highlight the need for standardized imaging protocols and shared calibration practices to improve inter-institutional reproducibility.

### ClearRT (kVCT)

This survey revealed notable inter-institutional variability in the creation and QA of CT–MD tables using ClearRT. Although the majority of respondents used Sun Nuclear phantoms, the inclusion of CIRS and Gammex models may have contributed to variability. In contrast, over 83% of institutions employed Gammex plugs specific to tomotherapy, indicating greater consistency in plug selection.

CT–MD tables were registered at system commissioning in >60% of institutions, whereas others completed registration during clinical use. Approximately half of the institutions reported no routine QA procedures, underscoring the need for a standardized QA framework—particularly given the impact of CT–MD accuracy on dose recalculation in ART.

More than half of the institutions adopted the half-phantom method for CT number acquisition, which was associated with improved reproducibility and reduced susceptibility to artifacts. Although SD s decreased for all plugs, the improvement was modest for low-density materials such as LN-450 lung and true water (Table 3), indicating that the benefit of the half-phantom configuration is density-dependent and becomes more pronounced for high-density plugs. However, some institutions continued to use the full-phantom method or selected the approach based on scanning protocol, indicating a lack of consensus regarding phantom configuration.

Evaluation using the ‘Thorax FOV 440 mm, Normal’ protocol demonstrated minimal inter-institutional variation in low-density regions (air, water, lung), supporting the reliability of this protocol for clinical dose calculations. In contrast, substantial deviations exceeding 300 HU were observed in high-density regions (e.g. CB-50%, cortical bone), likely reflecting differences in phantom materials, reconstruction methods and ROI settings. CT numbers for high-density plugs were generally higher with the half-phantom method, with differences reaching up to +270 HU. Based on the data presented in this study, the largest HU deviation observed in cortical bone plugs was approximately +300 HU. This corresponds to an MD difference of approximately +0.1 to +0.2 g/cm^3^. According to Kilby *et al.* (2005), a + 0.055 increase in electron density—roughly equivalent to a + 0.1 to +0.15 g/cm^3^ increase in bone MD—resulted in a −2.0% dose error for a 10 cm-thick cortical bone slab at 6 MV [15]. Extrapolating this relationship, a +0.2 g/cm^3^ MD deviation could lead to a dose error of −4% or more. This estimate suggests that HU deviations in high-density regions, such as bone, may lead to non-negligible dose calculation errors in ART, highlighting the need for precise CT-to-density calibration.

Although previous studies have shown that CT number uncertainties up to ±120 HU result in dose errors within 2–3%, further validation is necessary to determine the clinical significance of the observed variation [23]. In high-precision radiotherapy, such discrepancies may still be clinically relevant, particularly in heterogeneous anatomical regions. The half-phantom method also demonstrated smaller SDs, likely due to reduced susceptibility to air gaps and scan-related artifacts. Inaccuracies in CT numbers for high-density materials can introduce systematic dose calculation errors, potentially compromising target coverage and increasing exposure to organs at risk (OARs), thereby undermining overall treatment precision.

Additionally, differences in the physical properties of calibration phantoms contribute to inter-institutional variability. For example, the Tomo Medical Imaging Phantom has an MD of 1.047 g/cm^3^, whereas the Sun Nuclear Clear Virtual Water Phantom has a slightly lower density of 1.032 g/cm^3^. Although both are marketed as water-equivalent, their differences in MD and x-ray attenuation may cause measurable deviations in CT numbers for air and water, affecting CT–MD scaling and ultimately dose calculation accuracy.

These findings underscore the need for standardization not only in imaging and analysis protocols but also in the selection and handling of calibration phantoms. Overall, this study provides foundational insights into the current variability in CT–MD table creation and management using ClearRT. The adoption of the half-phantom method—particularly for high-density calibration—is recommended, in conjunction with standardized acquisition and analysis procedures. While the half-phantom configuration effectively minimizes beam hardening and scatter effects in ClearRT’s kVCT geometry, its direct application to SimCT presents practical limitations. SimCT systems are diagnostic-grade CT scanners with larger bore diameters and beam profiles optimized for full-phantom imaging. As such, standard phantoms and plug assemblies are not designed for split-phantom setups. Moreover, SimCT scanners typically incorporate vendor-specific correction algorithms for beam hardening and scatter during image reconstruction, and the effectiveness of these corrections may vary between systems. Implementing a half-phantom configuration on SimCT could introduce air gaps and geometric inconsistencies that degrade image uniformity, and may also interact unpredictably with existing correction processes. Therefore, extending the half-phantom method to SimCT would require redesigning the calibration phantom, verifying CT number accuracy and integrating the approach into existing QA workflows.

The half-phantom method is not currently endorsed by major AAPM guidelines (TG-66, TG-148, TG-306) and therefore remains a local practice [17, 21, 22]. In our survey, 28% of institutions reported adopting this configuration based on recommendations in the Accuray Precision Treatment Planning System manual. To date, its effectiveness has been demonstrated only with the Sun Nuclear SN-SW-Tomo phantom for ClearRT kVCT, and its applicability to other commercial phantom models or vendors has not been evaluated. Further studies should investigate this method across different phantom designs and consider its potential inclusion in national or international QA guidelines.

### CTrue (MVCT)

For calibration phantoms, the sun nuclear virtual water tomo phantom was used in 70% of the institutions. In terms of density plugs, 94% used tomotherapy-specific plugs from Gammex, suggesting the widespread adoption of dedicated equipment and a relatively standardized process. FBP was the most frequently used reconstruction algorithm (58%), followed by IR general (21%) and IR soft tissue (9%).

A comparison of CT numbers across multiple plugs (LN-300, LN-450, True water, CB-30%, CB-50%, Cortical bone) revealed no significant differences in mean values or SDs between FBP and IR, indicating stable results for both methods. Although some cases showed smaller variability with IR, the differences were inconsistent, suggesting that the impact of reconstruction method was minimal.

As shown in Fig. 2C, inter-facility variability in CTrue-derived CT–MD tables was smaller than that observed in SimCT or ClearRT, particularly in high-density regions, where strong agreement among institutions was observed. CT number variation for each density plug remained within AAPM TG-148 tolerance levels (±50 HU for bone, ±30 HU for water, ±50 HU for lung), implying that resulting dose errors would be within 2% [21]. Although all measured values met the TG-148 tolerance limits used for intra-institutional QA, it should be noted that these criteria were not designed for inter-institutional comparisons. Therefore, compliance with TG-148 limits at each site does not necessarily indicate minimal variability across institutions, particularly for materials such as air plugs that are not explicitly covered by the guideline [21]. These findings suggest that CT–MD tables based on CTrue provide sufficient accuracy for both clinical dose calculation and heterogeneity correction, and that the observed inter-institutional variability may fall within clinically acceptable limits.

However, CT numbers for air showed greater inter-institutional variation than other plugs. This may be attributed to differences in where air CT numbers are obtained during CT–MD table construction—specifically, whether measurements are taken inside or outside the phantom—as CT numbers can differ between these locations. Although air should ideally yield a CT number near −1000 HU, some institutions showed deviations up to ±30 HU, which could cause dose differences of up to ~1% [16]. These observations underline the need for harmonized measurement protocols—particularly for air—and suggest that future inter-institutional audits using a common calibration phantom would further enhance calibration consistency.

Regarding QA, 43% of the institutions reported not performing regular checks. Although this may seem problematic, many institutions used only ClearRT and did not use CTrue for dose calculation; therefore, QA for the CTrue CT–MD table was not performed. Overall, the CT–MD tables constructed using CTrue demonstrated high accuracy and inter-facility consistency. The influence of the reconstruction algorithm choice was minor, and the dose calculations based on these tables appeared robust. Further improvements are expected through the standardization of air CT number acquisition and the implementation of regular QA in institutions utilizing CTrue for clinical dose computation.

### Current status of data point distribution and proposal for standardization guidelines

In SimCT, the median number of calibration points was 9–10, with several institutions exceeding 20, indicating notable inter-institutional variability. ClearRT and CTrue showed narrower distributions, with medians of eight points. The use of plastic plugs with CT numbers near 0–100 HU is not recommended, as their physical density may not accurately represent real tissue. Instead, direct measurement of water to establish a reference at ~1.0 g/cm^3^ and 0 HU is advised [20]. This recommendation is based on vendor-provided technical guidance, reflecting calibration requirements specific to the Accuray Precision Treatment Planning System, which uses MD for all dose calculations. To our knowledge, this approach has not been formally evaluated or validated in peer-reviewed literature, and this limitation is noted in the present study.

These findings suggest that standardized procedures for constructing CT–MD tables are not uniformly implemented across institutions. Differences in table resolution and shape may stem from variations in phantom materials, calibration protocols or institutional priorities—for example, between facilities operating both linear accelerators and tomotherapy systems versus those using tomotherapy alone. Although the half-phantom method has been adopted by some institutions and offers practical advantages, current evidence is insufficient to definitively recommend one calibration method over another. Similarly, the optimal number of data points remains undetermined, and its relationship with dose calculation accuracy requires further investigation.

Therefore, national standardization efforts may be better focused on three key areas: phantom acquisition geometry, air CT-number measurement and reconstruction documentation. These recommendations, together with suggested QA frequencies based on AAPM TG-148 and TG-306, are summarized in Table 5.

**Table 5:** Recommendations for ClearRT and CTrue

Topic	Current issue	Proposed solution	Recommended QA frequency^*^
Phantom setup (ClearRT, kVCT)	Large inter-institutional variability in high-density plugs; differences in setup methods	Use a half-phantom when acquiring the CT–MD table	At commissioning; periodically (e.g. quarterly or monthly if used for ART dose recalculation); after any imaging/reconstruction change
Air CT-number measurement (CTrue, MVCT)	Values vary by measurement location; inter-institutional drift up to ±30 HU	Measure bore air outside the phantom; fix measurement location; document the method; target ≈ −1000 HU with an internal action level of ±30 HU	Periodically (e.g. quarterly, or monthly if used for ART dose recalculation); spot-check after protocol changes or suspected drift
Reconstruction documentation (CTrue, MVCT)	Insufficient parameter recording hinders reproducibility	Record and lock the algorithm type (FBP/IR and IR level); save as a protocol; record any changes	At commissioning; whenever parameters change; annual documentation audit

^*^QA frequencies are based on AAPM TG-148 and TG-306 and should be adapted to institutional policy and vendor instructions.

With the growing implementation of ART, the QA of inhomogeneity correction tables used in on-board systems is expected to play a critical role in maintaining treatment accuracy and patient safety. The present results provide foundational data to support the development of a robust QA/QC framework tailored to emerging clinical demands.

A limitation of this study is that, while inter-institutional variability in CT–MD table construction and registration was characterized, its dosimetric impact was not evaluated. In addition, a common calibration phantom was not employed across institutions, which prevented objective verification of the absolute accuracy of individual CT–MD tables; inter-institutional audits using a standardized phantom will therefore be required. Moreover, because the survey relied on self-reported data, the objectivity and accuracy of the responses may be limited. Further research is needed to quantify the clinical consequences of this variability and to validate reported practices through objective measurements.

## CONCLUSION

This study investigated the construction and implementation of CT–MD tables for SimCT, ClearRT and CTrue across 34 Radixact-equipped institutions in Japan. For SimCT, CT numbers for water and air generally fell within the AAPM TG-66 tolerance; however, deviations exceeding 300 HU were observed in high-density regions. For ClearRT, the half-phantom method improved reproducibility, although variability remained dependent on imaging conditions and phantom setup. CTrue demonstrated consistent CT numbers across institutions, with all values within AAPM TG-148 tolerance limits. Nevertheless, air CT numbers showed variation of up to ±50 HU.

These findings underscore the need to standardize imaging protocols, CT–MD table construction methods and routine QA practices to ensure consistent and accurate dose calculations—particularly as adaptive radiotherapy becomes more widely implemented in clinical practice.

## Supplementary Material

SupplementaryData_modify_rraf063

## PRESENTATION AT A CONFERENCE

Portions of this work were presented at a national academic meeting, but the complete study has not been previously presented or published.
