# Supplementary material for: Inter-institutional variability in CT-to-mass-density conversion tables for helical tomotherapy: a national survey in Japan
Source: J Radiat Res. 2025 Oct 16;66(6):606–16. doi: 10.1093/jrr/rraf063 (PMC12648070; doi:10.1093/jrr/rraf063)
Supplement: SupplementaryData_modify_rraf063 [file supplementarydata_modify_rraf063.docx]

**Supplementary Data**

This supplementary material provides a detailed comparison of CT-to-mass density conversion tables (CT–MD tables) constructed and registered in the Precision treatment planning system across multiple institutions.

Figure A presents the CT–MD tables obtained using the ClearRT imaging system under ten different scanning protocols. Each figure shows the relationship between CT number (HU) and physical mass density (g/cm³) (n=27-29).

Figure B shows CT–MD tables constructed using simulation CT (n = 34).

Figure C displays CT–MD tables derived from MVCT (n = 34).

These data highlight the inter-institutional and modality-based variability in CT–MD mapping, which may influence the accuracy of dose calculations, particularly in adaptive radiation therapy.

**
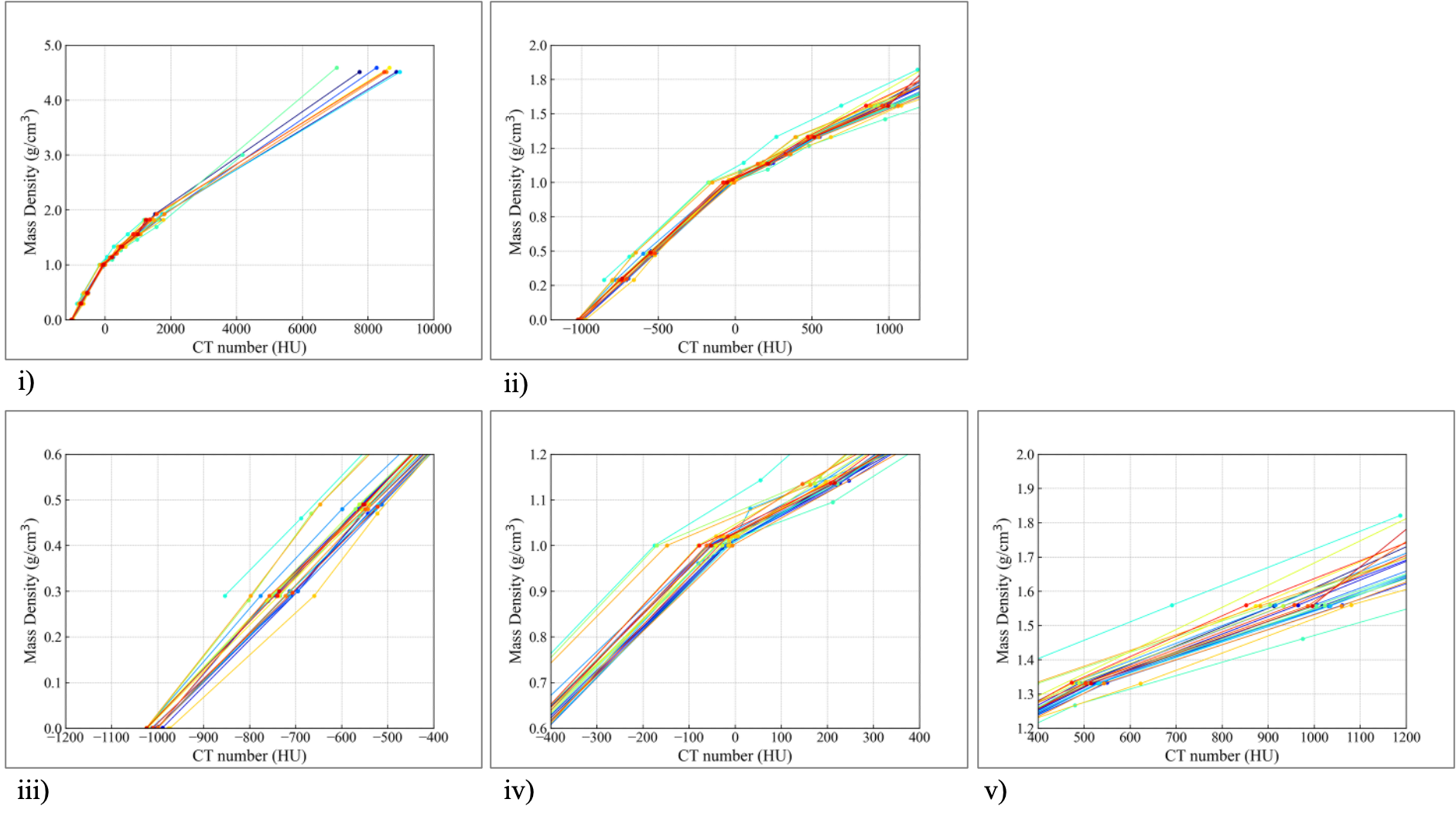
**

Figure A1. CT-MD Table for Head FOV 270 mm (Fine) acquired using ClearRT and registered in Precision (n=27).

Each panel shows CT number–to–mass density (CT-MD) calibration curves obtained from multiple institutions using ClearRT.
For the scan condition Head FOV 270 mm (Fine), five subpanels illustrate the calibration curves across the following mass density regions of interest:
(i) full range (0–5.0 g/cm³),(ii) soft tissue to bone (0–2.0 g/cm³),(iii) lung-equivalent region (0.1–0.6 g/cm³),(iv) water-equivalent region (0.7–1.2 g/cm³), and　(v) bone-equivalent region (1.2–2.4 g/cm³).


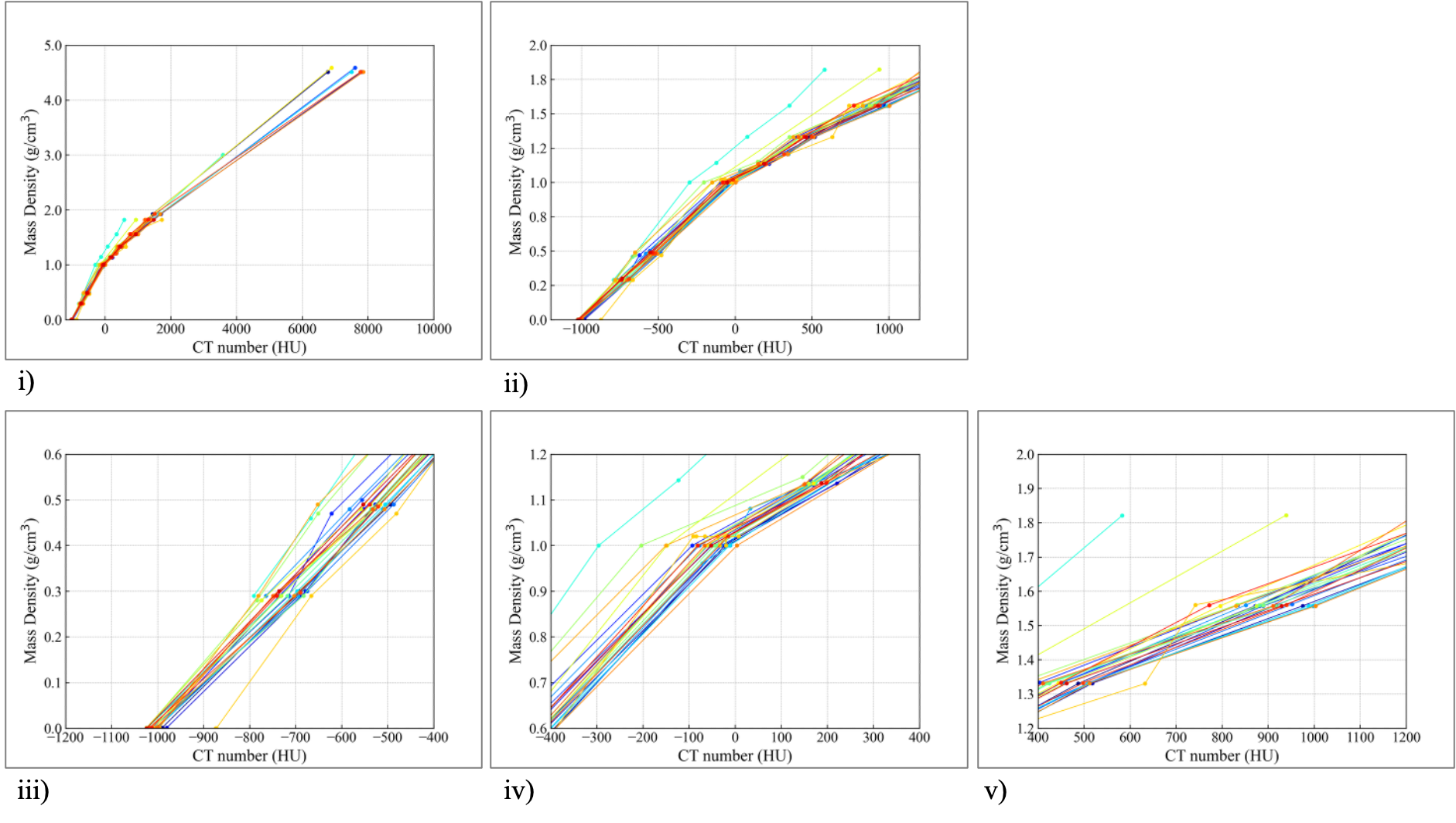


Figure A2. CT-MD Table for Head FOV 270 mm (Normal) acquired using ClearRT and registered in Precision (n=27).

Each panel shows CT number–to–mass density (CT-MD) calibration curves obtained from multiple institutions using ClearRT.
For the scan condition Head FOV 270 mm (fine), five subpanels illustrate the calibration curves across the following mass density regions of interest:
Five subpanels correspond to the same mass density regions defined in **Figure 1A**.


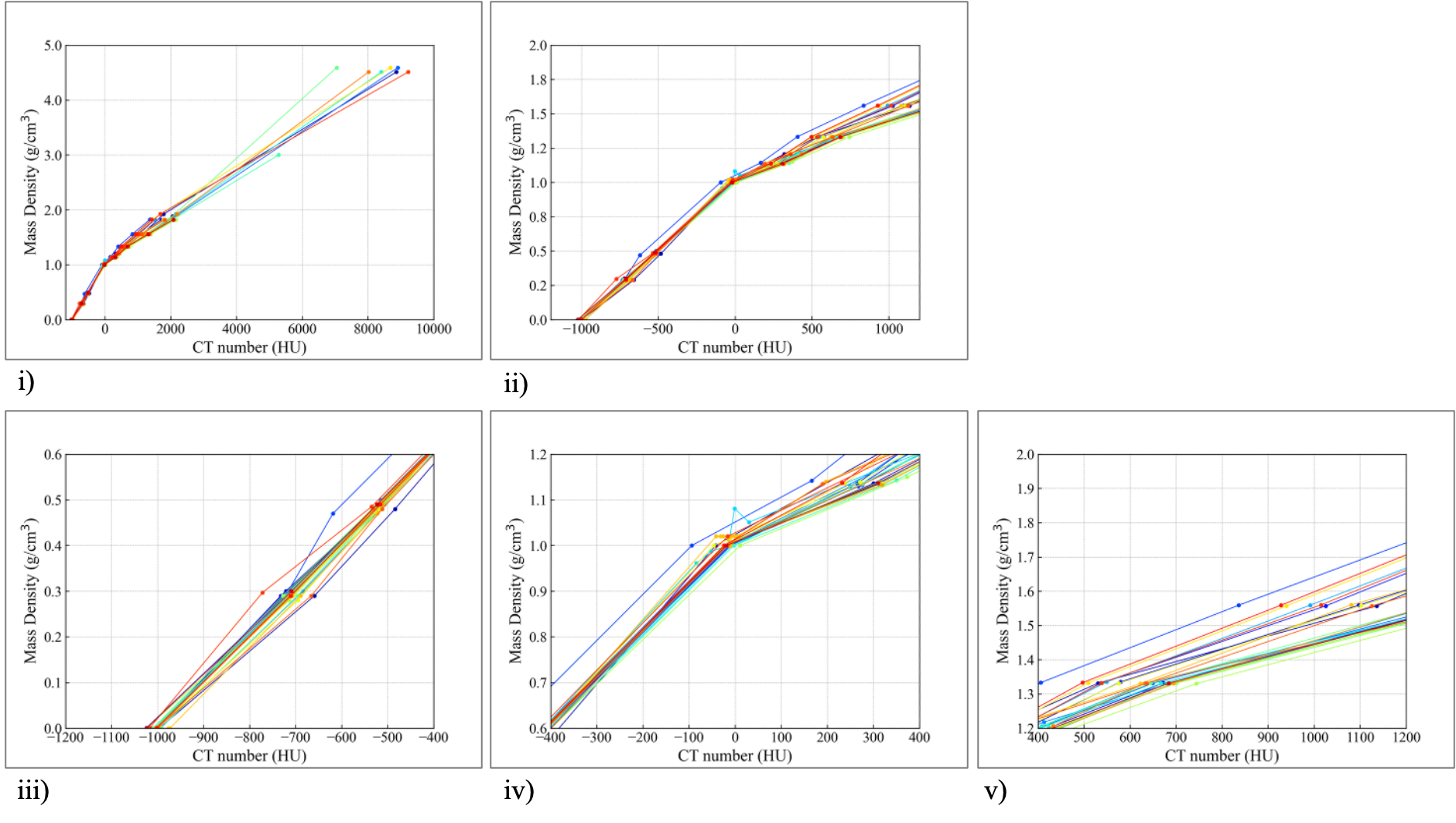


Figure A3. CT-MD Table for Head FOV 440 mm (Fine) acquired using ClearRT and registered in Precision (n=28).

Each panel shows CT number–to–mass density (CT-MD) calibration curves obtained from multiple institutions using ClearRT.
For the scan condition Head FOV 440 mm (Fine), five subpanels illustrate the calibration curves across the following mass density regions of interest:
Five subpanels correspond to the same mass density regions defined in **Figure 1A**.


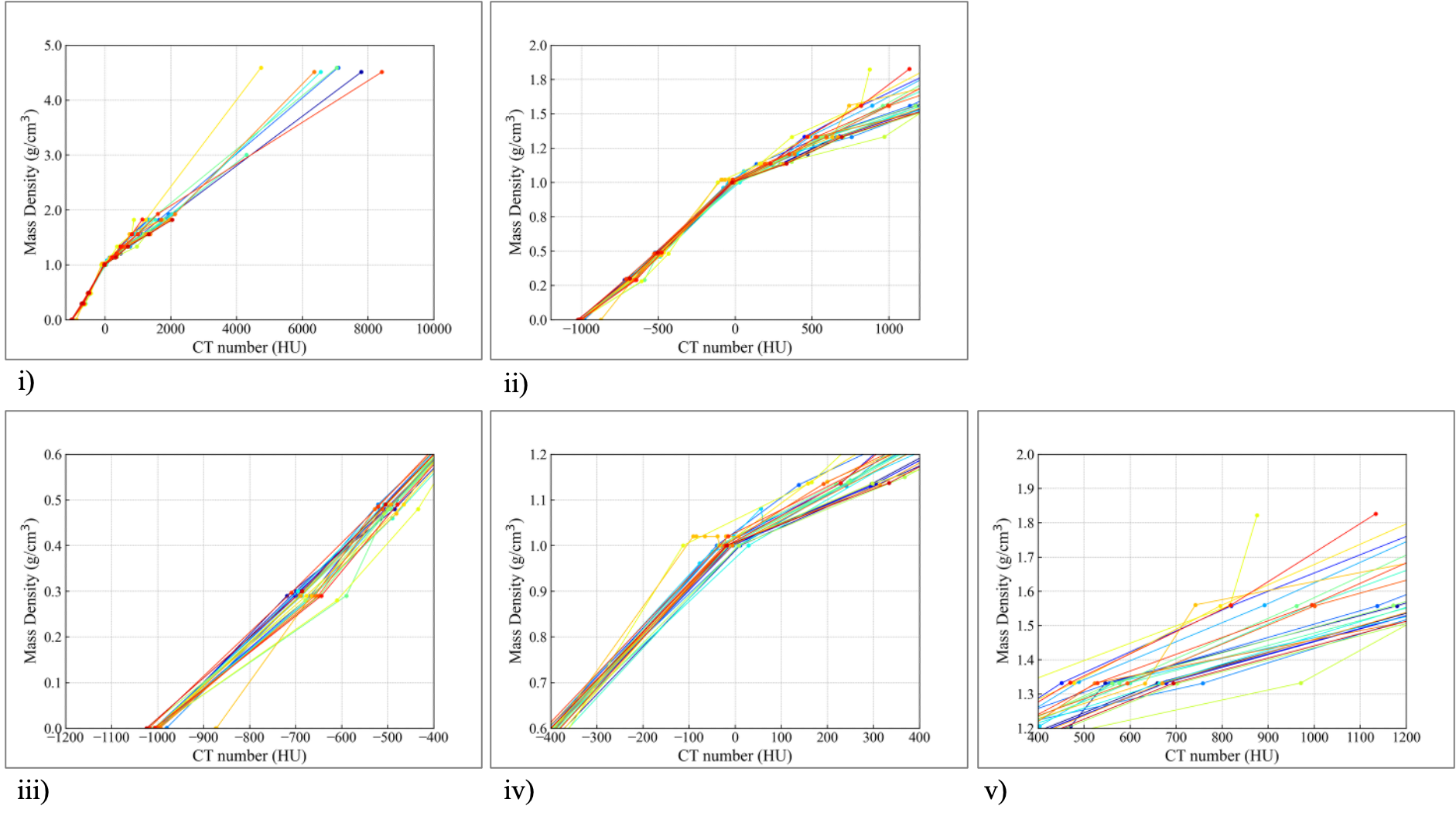


Figure A4. CT-MD Table for Head FOV 440 mm (Normal) acquired using ClearRT and registered in Precision (n=28).

Each panel shows CT number–to–mass density (CT-MD) calibration curves obtained from multiple institutions using ClearRT.
For the scan condition Head FOV 440 mm (Normal), five subpanels illustrate the calibration curves across the following mass density regions of interest:
Five subpanels correspond to the same mass density regions defined in **Figure 1A**.

**
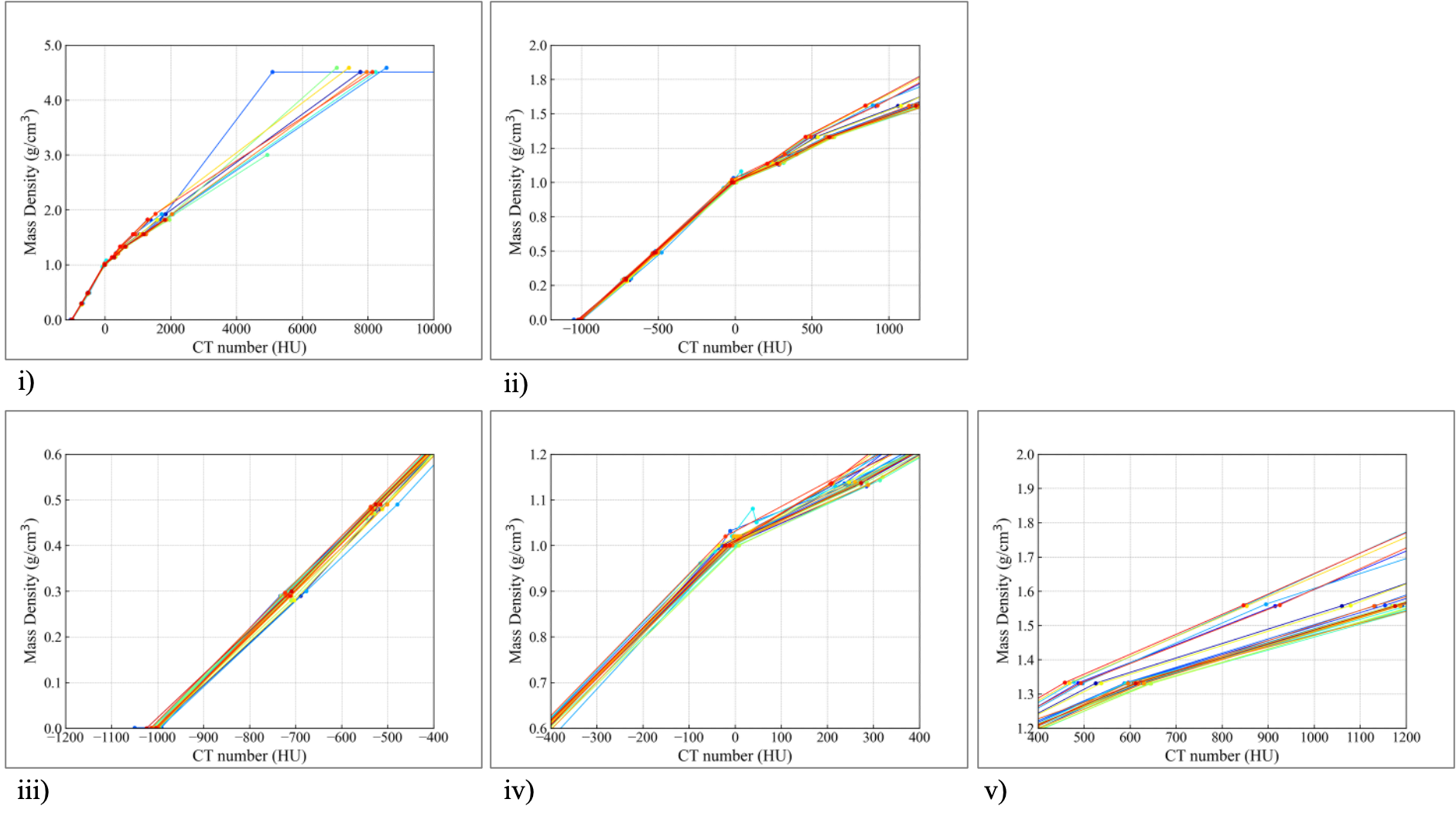
**

Figure A5. CT-MD Table for Thorax FOV 440 mm (Fine) acquired using ClearRT and registered in Precision (n=29).

Each panel shows CT number–to–mass density (CT-MD) calibration curves obtained from multiple institutions using ClearRT.
For the scan condition Thorax FOV 440 mm (Fine), five subpanels illustrate the calibration curves across the following mass density regions of interest:
Five subpanels correspond to the same mass density regions defined in **Figure 1A**.

**
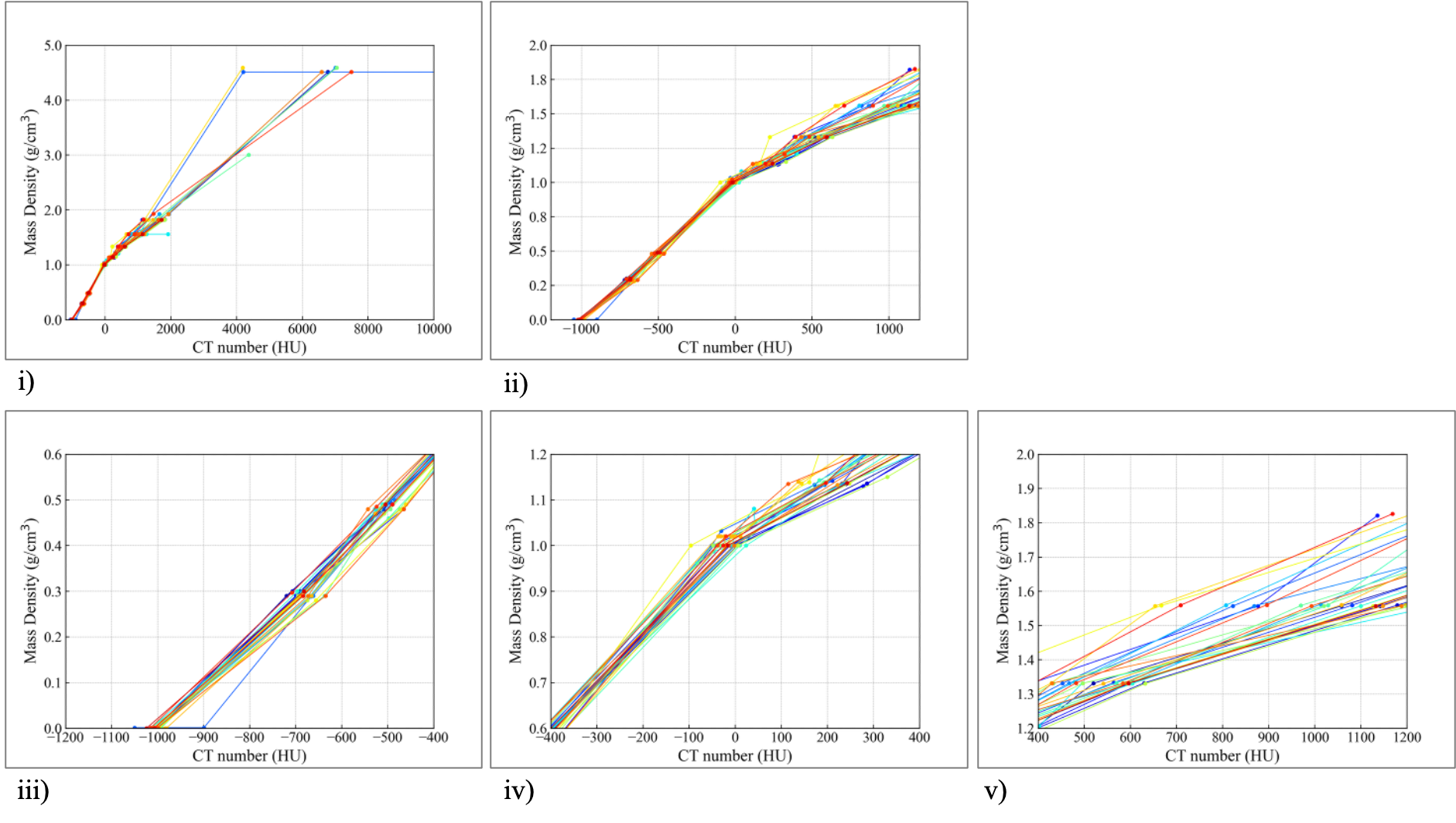
**

Figure A6. CT-MD Table for Thorax FOV 440 mm (Normal) acquired using ClearRT and registered in Precision (n=29).

Each panel shows CT number–to–mass density (CT-MD) calibration curves obtained from multiple institutions using ClearRT.
For the scan condition Thorax FOV 440 mm (Normal), five subpanels illustrate the calibration curves across the following mass density regions of interest:
Five subpanels correspond to the same mass density regions defined in **Figure 1A**.


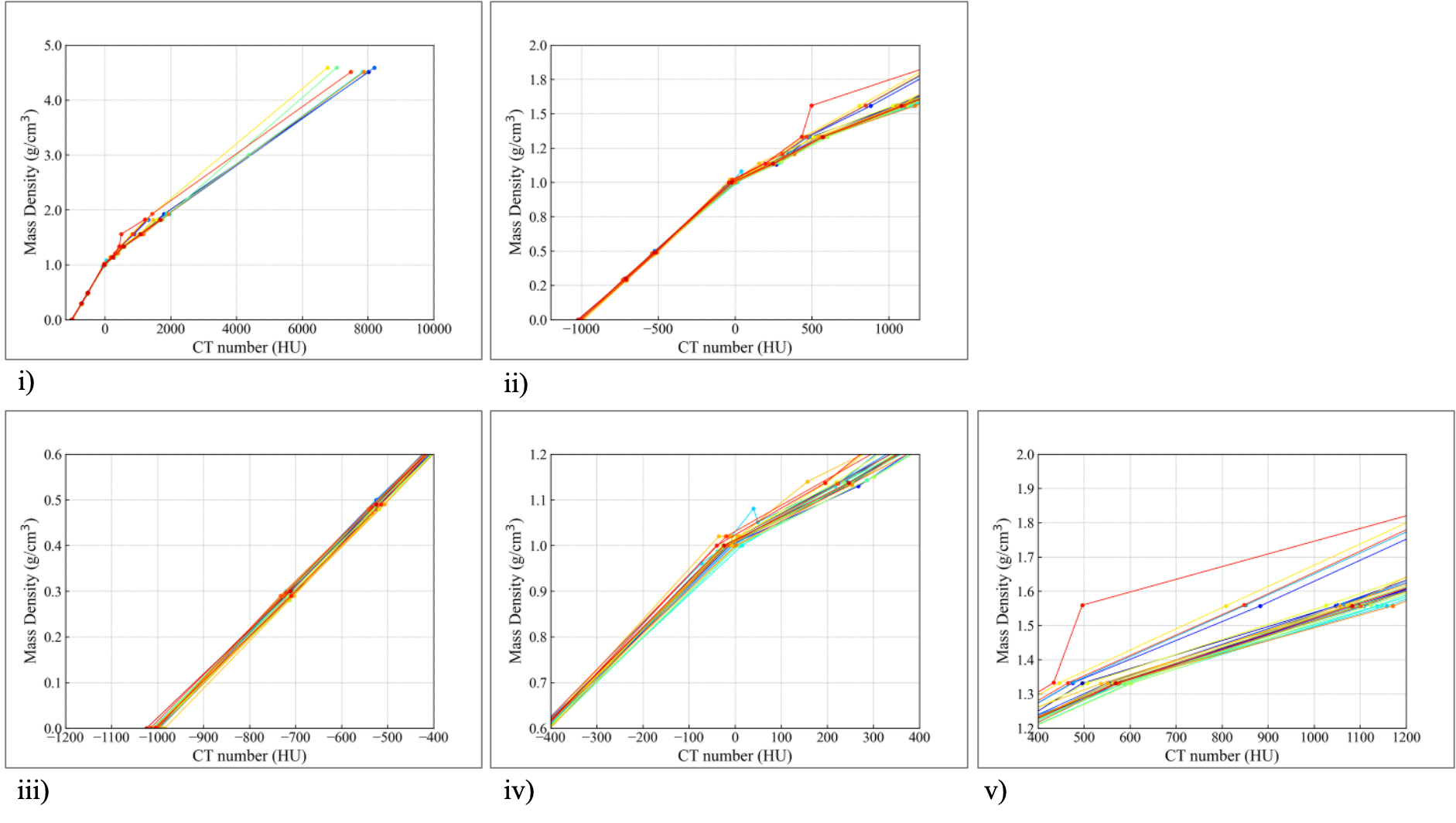


Figure A7. CT-MD Table for Pelvis FOV 440 mm (Fine) acquired using ClearRT and registered in Precision (n=28).

Each panel shows CT number–to–mass density (CT-MD) calibration curves obtained from multiple institutions using ClearRT.
For the scan condition Pelvis FOV 440 mm (Fine), five subpanels illustrate the calibration curves across the following mass density regions of interest:
Five subpanels correspond to the same mass density regions defined in **Figure 1A**.

**
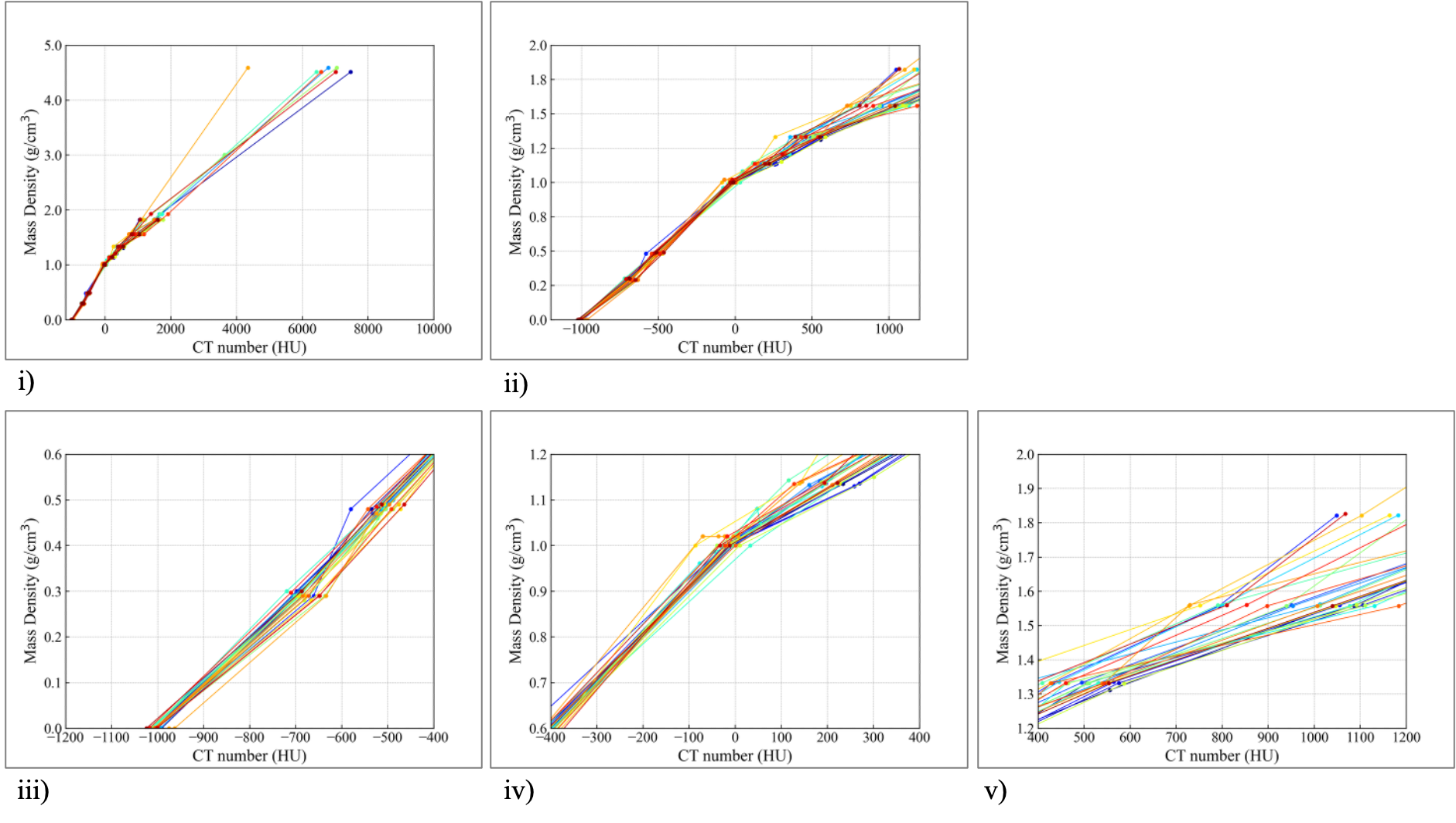
**

Figure A8. CT-MD Table for Pelvis FOV 440 mm (Normal) acquired using ClearRT and registered in Precision (n=28).

Each panel shows CT number–to–mass density (CT-MD) calibration curves obtained from multiple institutions using ClearRT.
For the scan condition Pelvis FOV 440 mm (Normal), five subpanels illustrate the calibration curves across the following mass density regions of interest:
Five subpanels correspond to the same mass density regions defined in **Figure 1A**.


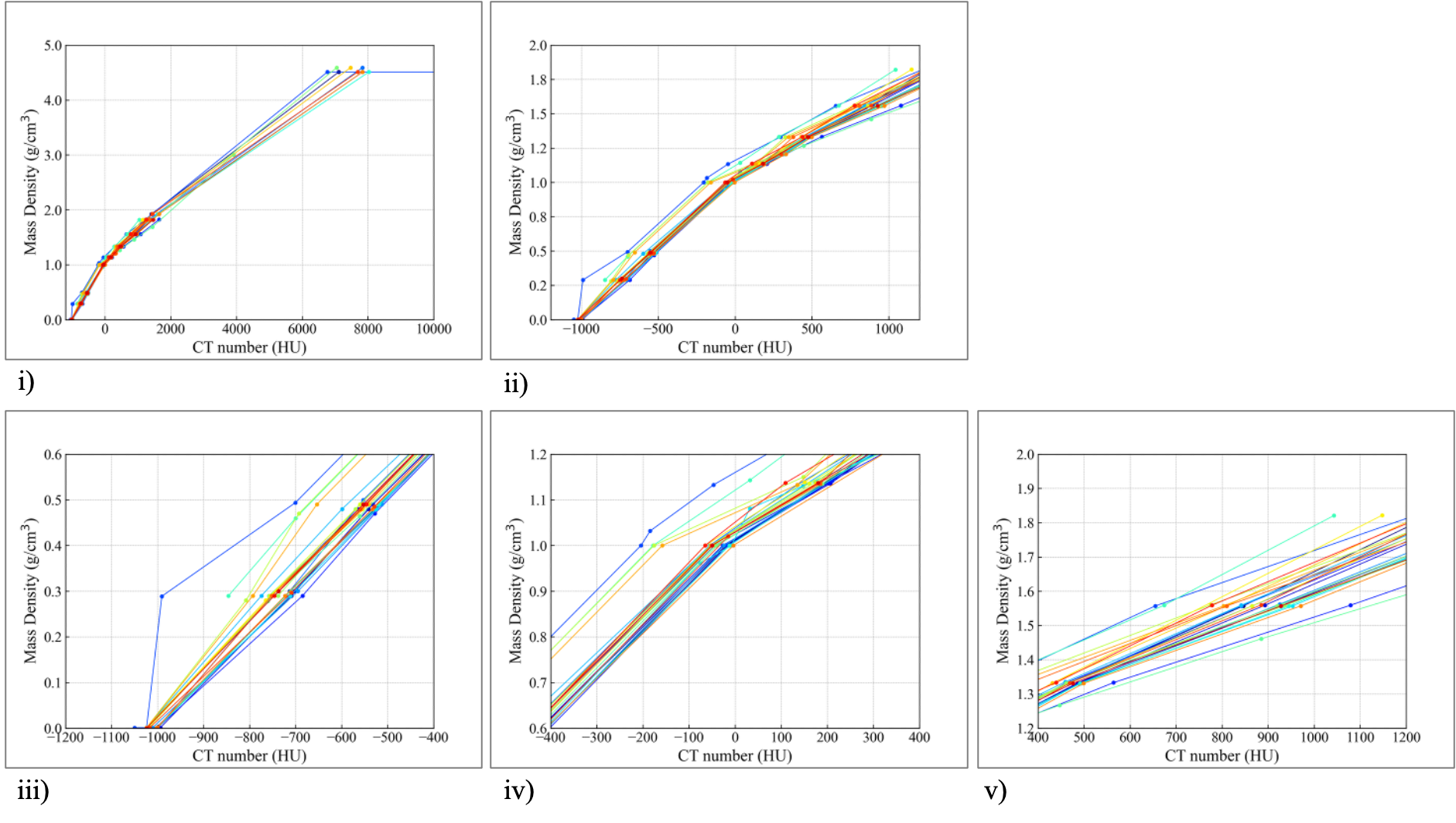


Figure A9. CT-MD Table for Whole Body FOV 270 mm (Fine) acquired using ClearRT and registered in Precision (n=27).

Each panel shows CT number–to–mass density (CT-MD) calibration curves obtained from multiple institutions using ClearRT.
For the scan condition Whole Body FOV 270 mm (Fine), five subpanels illustrate the calibration curves across the following mass density regions of interest:
Five subpanels correspond to the same mass density regions defined in **Figure 1A.**


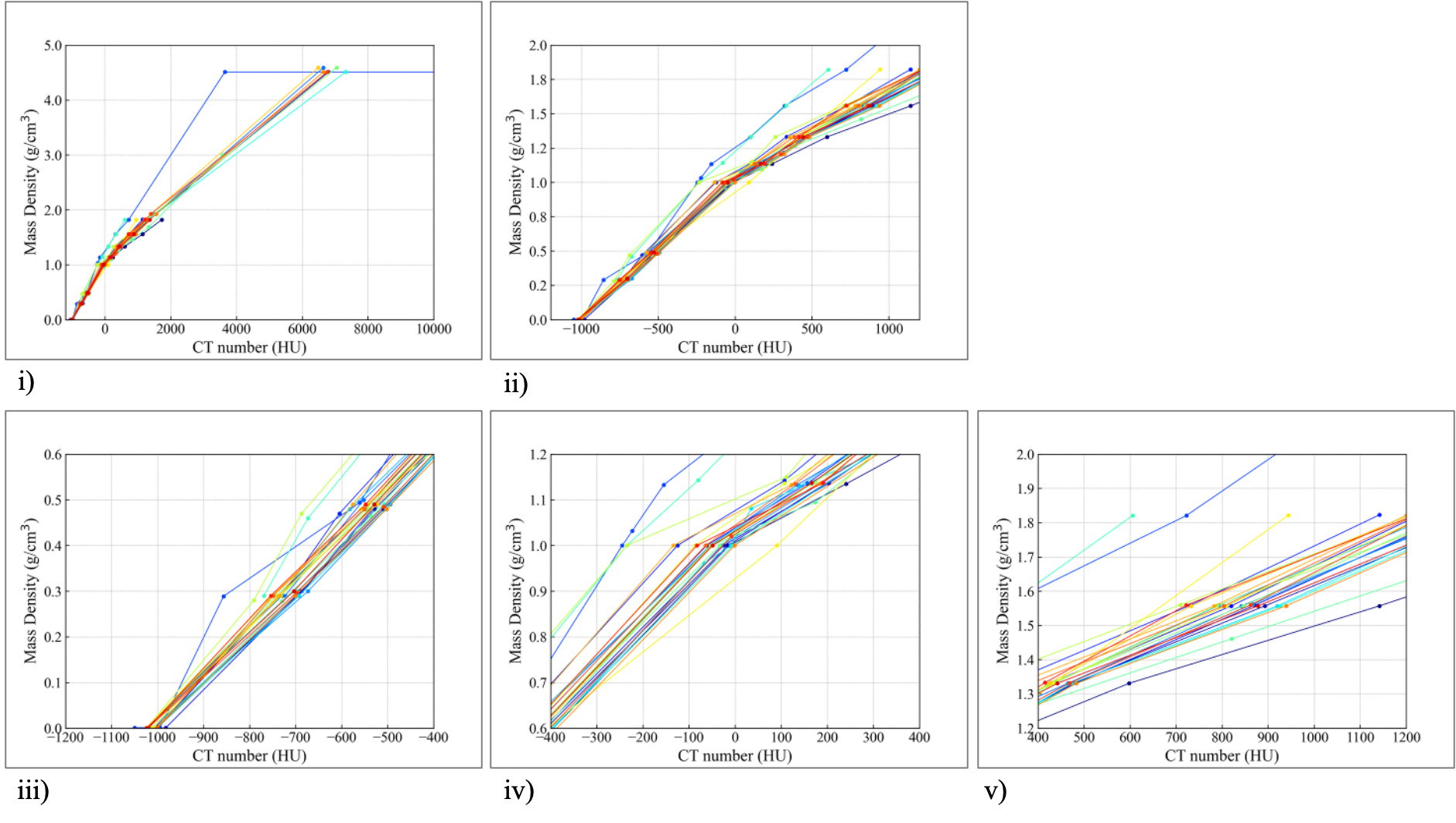


Figure A10. CT-MD Table for Whole Body FOV 270 mm (Fine) acquired using ClearRT and registered in Precision (n=27).

Each panel shows CT number–to–mass density (CT-MD) calibration curves obtained from multiple institutions using ClearRT.
For the scan condition Whole Body FOV 270 mm (Fine), five subpanels illustrate the calibration curves across the following mass density regions of interest:
Five subpanels correspond to the same mass density regions defined in**Figure 1A.**

**
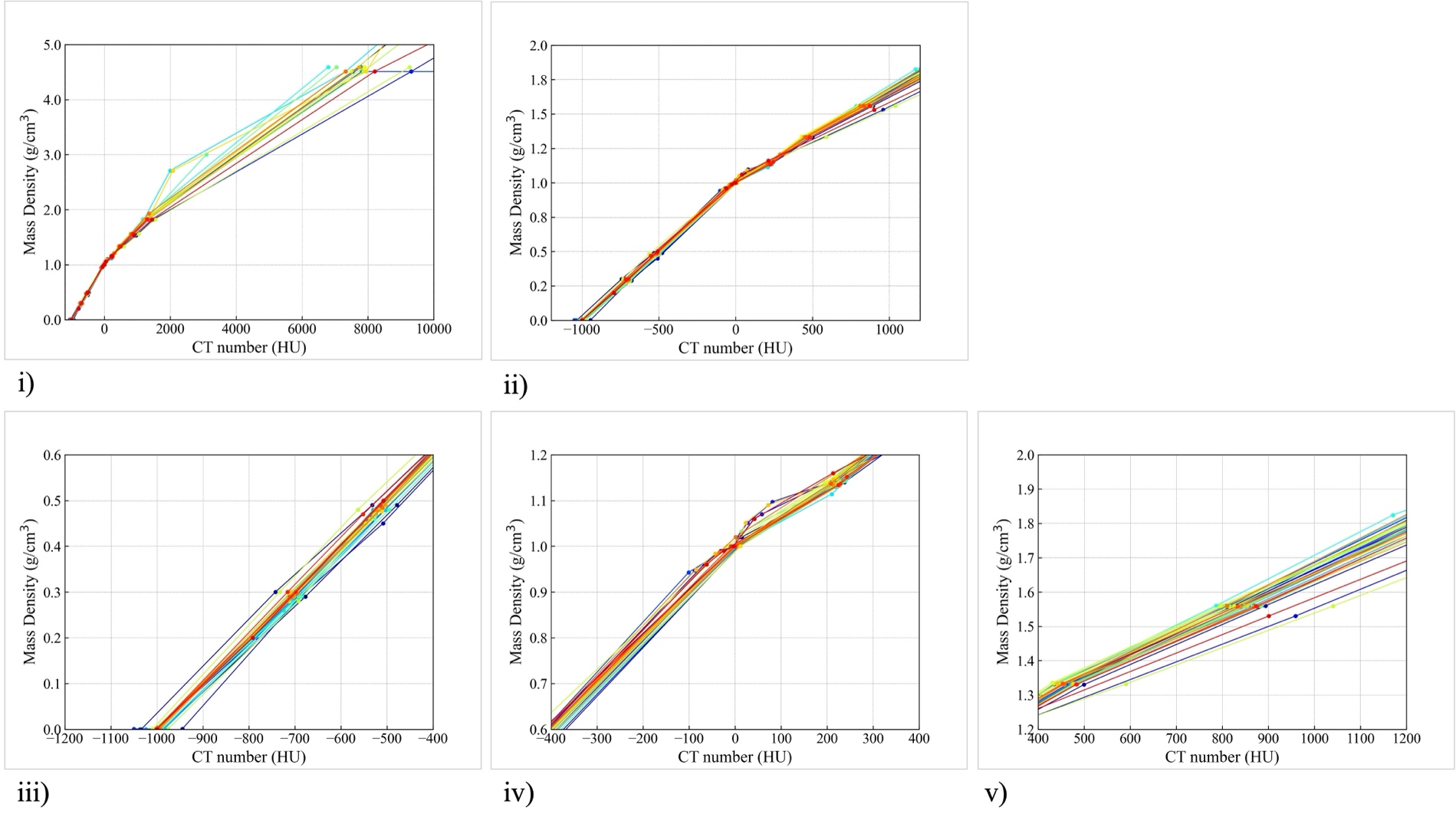
**

Figure B. CT–MD tables for simulation CT registered in Precision (n = 34).
Each panel shows CT number–to–mass density (CT–MD) calibration curves collected from multiple institutions using simulation CT.
The five subpanels illustrate calibration curves across the same mass density regions of interest defined in Figure 1A.

**
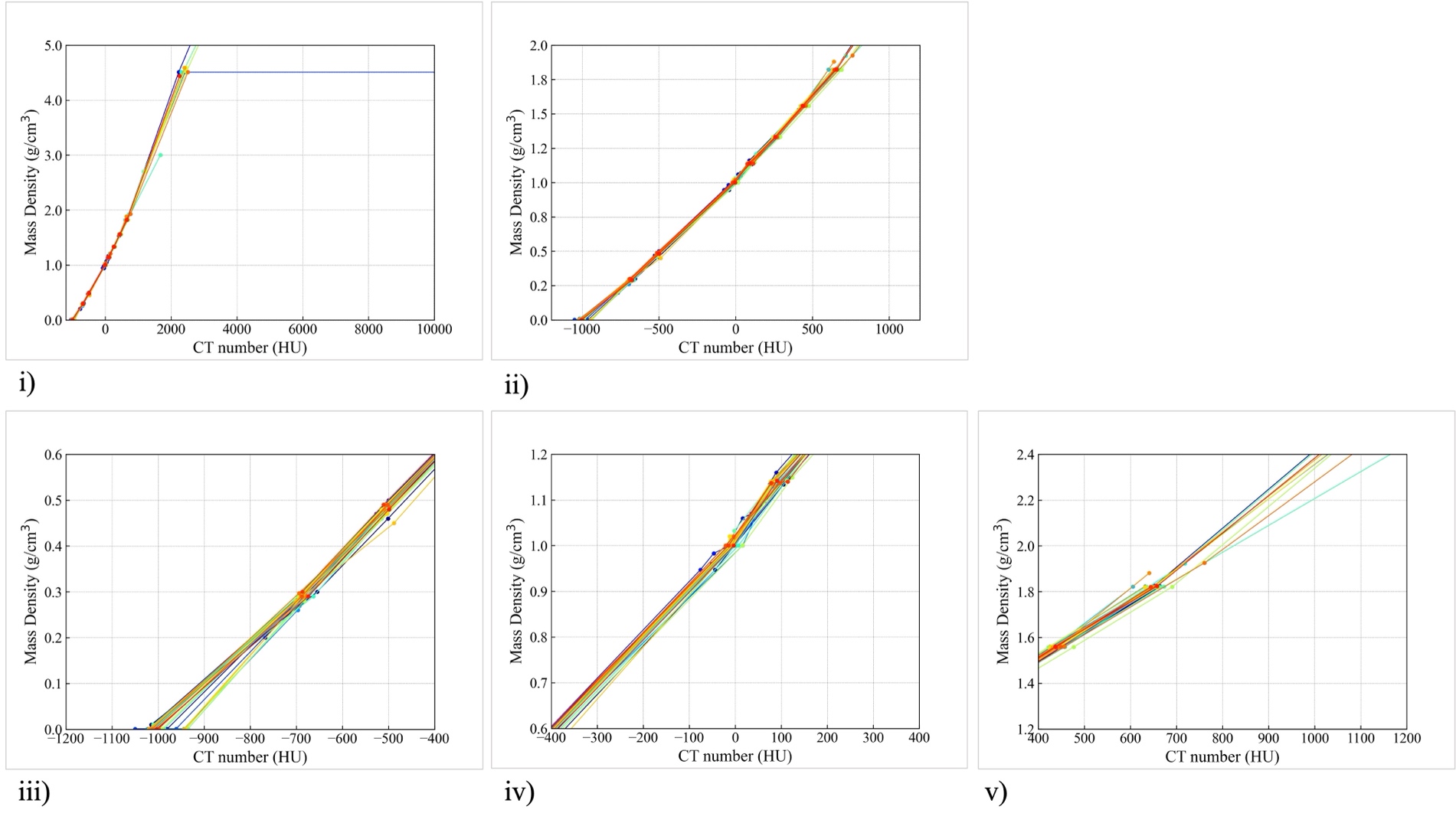
**

Figure C. CT–MD tables for MVCT registered in Precision (n = 34).
Each panel shows CT number–to–mass density (CT–MD) calibration curves collected from multiple institutions using MVCT.
The five subpanels illustrate calibration curves across the same mass density regions of interest defined in Figure 1A.

**Supplementary Data Table A. Inter-Facility Comparison of CT–MD Tables Generated with Full- and Half-Phantom Methods, Including Combined Dataset**

This table presents a comparison of CT number (MD) for eight tissue-equivalent density plugs used in CT–MD tables registered in Precision. The CT values were derived from ClearRT scans acquired using two different calibration methods: the full-phantom method and the half-phantom method. In addition, a combined dataset incorporating all available data is included for comprehensive comparison.

Legend (Tables A1–A10). For each tissue-equivalent plug, the mass density (MD) is given as mean (Min–Max), and the CT number as mean ± 1 SD. Three data sources are shown: full-phantom, half-phantom, and the combined dataset. *n indicates the number of participating facilities. † Data from a single institution; SD not applicable.

Table A1. Comparison of CT number for eight tissue-equivalent density plugs used in CT–MD tables registered in Precision, constructed from ClearRT Head FOV 270 mm (Fine) images using full-phantom and half-phantom methods, as well as the combined dataset.

|  |  | Entire data | |  | Full phantom method | |  | Half phantom method | |
| --- | --- | --- | --- | --- | --- | --- | --- | --- | --- |
|  |  | MD (g/cm^3^) | CT number (HU) |  | MD (g/cm^3^) | CT number (HU) |  | MD (g/cm^3^) | CT number (HU) |
| Tissue-equivalent  density plug | n* | Mean (Min-Max) | Mean ± 1SD | n* | Mean (Min-Max) | Mean ± 1SD | n* | Mean (Min-Max) | Mean ± 1SD |
| Air | 18 | 0.001 (0.001 – 0.001) | -1009.7 ± 12.2 | 9 | 0.001 (0.001 – 0.001) | -1010.3 ± 11.2 | 9 | 0.001 (0.001 – 0.001) | -1009.1 ± 13.7 |
| LN-300 Lung | 27 | 0.29 (0.28 – 0.31) | -742.1 ± 43.4 | 15 | 0.29 (0.28 – 0.31) | -746.1 ± 53.4 | 12 | 0.29 (0.28 – 0.30) | -737.1 ± 28.0 |
| LN-450 Lung | 27 | 0.48 (0.45 – 0.50) | -562.6 ± 48.1 | 15 | 0.48 (0.45 – 0.49) | -568.6 ± 57.7 | 12 | 0.49 (0.46 – 0.50) | -555.2 ± 33.6 |
| True water | 27 | 1.00 (1.00 – 1.02) | -55.3 ± 52.4 | 15 | 1.00 (1.00 – 1.02) | -63.2 ± 63.0 | 12 | 1.00 (1.00 – 1.02) | -45.5 ± 35.5 |
| Inner bone | 25 | 1.16 (1.13 – 1.22) | 233.3 ± 89.7 | 14 | 1.16 (1.13 – 1.22) | 217.9 ± 111.5 | 11 | 1.15 (1.13 – 1.21) | 230.2 ± 55.2 |
| CB-30 % | 25 | 1.33 (1.33 – 1.34) | 486.7 ± 77.8 | 14 | 1.33 (1.33 – 1.34) | 477.1 ± 98.3 | 11 | 1.33 (1.33 – 1.33) | 498.8 ± 41.0 |
| CB-50 % | 25 | 1.56 (1.56 – 1.56) | 950.4 ± 103.7 | 14 | 1.56 (1.56 – 1.56) | 937.7 ± 127.7 | 11 | 1.56 (1.56 – 1.56) | 966.6 ± 64.2 |
| Cortical bone (1.82 g/cm³) | 17 | 1.82 (1.82 – 1.83) | 1435.3 ± 172.0 | 8 | 1.82 (1.82 – 1.83) | 1394.9 ± 210.9 | 9 | 1.82 (1.82 – 1.82) | 1471.2 ± 131.1 |
| Cortical bone (1.92 g/cm³) | 8 | 1.92 (1.92 – 1.93) | 1685.5 ± 108.9 | 6 | 1.92 (1.92 – 1.93) | 1702.7 ± 100.9 | 2 | 1.92 (1.92 – 1.92) | 1633.9 ± 158.3 |

Table A2. Comparison of CT number for eight tissue-equivalent density plugs used in CT–MD tables registered in Precision, constructed from ClearRT Head FOV 270 mm (Normal) images using full-phantom and half-phantom methods, as well as the combined dataset.

|  |  | Entire data | |  | Full phantom method | |  | Half phantom method | |
| --- | --- | --- | --- | --- | --- | --- | --- | --- | --- |
|  |  | MD (g/cm^3^) | CT number (HU) |  | MD (g/cm^3^) | CT number (HU) |  | MD (g/cm^3^) | CT number (HU) |
| Tissue-equivalent  density plug | n* | Mean (Min-Max) | Mean ± 1SD | n* | Mean (Min-Max) | Mean ± 1SD | n* | Mean (Min-Max) | Mean ± 1SD |
| Air | 21 | 0.001 (0.001 – 0.001) | -1009.0 ± 12.7 | 11 | 0.001 (0.001 – 0.001) | -1012.6 ± 10.3 | 9 | 0.001 (0.001 – 0.001) | -1005.1 ± 14.4 |
| LN-300 Lung | 27 | 0.29 (0.28 – 0.31) | -729.9 ± 41.4 | 16 | 0.29 (0.28 – 0.31) | -732.9 ± 47.4 | 12 | 0.29 (0.29 – 0.30) | -722.3 ± 30.4 |
| LN-450 Lung | 27 | 0.48 (0.45 – 0.50) | -550.7 ± 53.9 | 16 | 0.48 (0.45 – 0.50) | -552.6 ± 59.2 | 12 | 0.49 (0.47 – 0.50) | -548.6 ± 45.8 |
| True water | 28 | 1.00 (1.00 – 1.02) | -71.2 ± 69.5 | 16 | 1.00 (1.00 – 1.02) | -78.6 ± 85.2 | 12 | 1.00 (1.00 – 1.00) | -59.6 ± 36.7 |
| Inner bone | 26 | 1.16 (1.13 – 1.22) | 20.53 ± 108.8 | 15 | 1.16 (1.13 – 1.22) | 206.8 ± 135.7 | 11 | 1.15 (1.13 – 1.21) | 200.5 ± 59.7 |
| CB-30 % | 25 | 1.33 (1.33 – 1.34) | 439.1 ± 95.3 | 14 | 1.33 (1.33 – 1.34) | 430.5 ± 123.2 | 11 | 1.33 (1.33 – 1.33) | 455.2 ± 44.2 |
| CB-50 % | 25 | 1.56 (1.56 – 1.56) | 866.1 ± 136.5 | 14 | 1.56 (1.56 – 1.56) | 854.4 ± 178.7 | 11 | 1.56 (1.56 – 1.56) | 881.7 ± 49.9 |
| Cortical bone (1.82 g/cm³) | 17 | 1.82 (1.82 – 1.83) | 1281.7 ± 217.5 | 8 | 1.82 (1.82 – 1.83) | 1187.3 ± 289.1 | 9 | 1.82 (1.82 – 1.82) | 1374.7 ± 70.4 |
| Cortical bone (1.92 g/cm³) | 8 | 1.92 (1.92 – 1.93) | 1610.5 ± 89.5 | 6 | 1.92 (1.92 – 1.93) | 1629.2 ± 69.4 | 2 | 1.92 (1.92 – 1.92) | 1544.6 ± 153.6 |

Table A3. Comparison of CT number for eight tissue-equivalent density plugs used in CT–MD tables registered in Precision, constructed from ClearRT Head FOV 440 mm (Fine) images using full-phantom and half-phantom methods, as well as the combined dataset.

|  |  | Entire data | |  | Full phantom method | |  | Half phantom method | |
| --- | --- | --- | --- | --- | --- | --- | --- | --- | --- |
|  |  | MD (g/cm^3^) | CT number (HU) |  | MD (g/cm^3^) | CT number (HU) |  | MD (g/cm^3^) | CT number (HU) |
| Tissue-equivalent  density plug | n* | Mean (Min-Max) | Mean ± 1SD | n* | Mean (Min-Max) | Mean ± 1SD | n* | Mean (Min-Max) | Mean ± 1SD |
| Air | 24 | 0.001 (0.001 – 0.001) | -1002.8 ± 6.8 | 12 | 0.001 (0.001 – 0.001) | -1001.5 ± 6.6 | 11 | 0.001 (0.001 – 0.001) | -1004.5 ± 7.2 |
| LN-300 Lung | 28 | 0.29 (0.28 – 0.31) | -708.1 ± 22.0 | 15 | 0.29 (0.28 – 0.31) | -699.9 ± 26.9 | 12 | 0.29 (0.28 – 0.30) | -718.4 ± 7.6 |
| LN-450 Lung | 28 | 0.48 (0.45 – 0.50) | -525.0 ± 21.6 | 15 | 0.48 (0.45 – 0.49) | -520.7 ± 14.0 | 12 | 0.49 (0.46 – 0.50) | -531.0 ± 28.7 |
| True water | 28 | 1.00 (1.00 – 1.02) | -22.2 ± 18.4 | 15 | 1.00 (1.00 – 1.02) | -20.9 ± 15.1 | 12 | 1.00 (1.00 – 1.02) | -25.3 ± 22.1 |
| Inner bone | 28 | 1.16 (1.13 – 1.22) | 302.3 ± 69.9 | 15 | 1.16 (1.13 – 1.22) | 309.6 ± 80.1 | 12 | 1.15 (1.13 – 1.21) | 283.3 ± 45.5 |
| CB-30 % | 27 | 1.33 (1.33 – 1.34) | 611.6 ± 86.2 | 14 | 1.33 (1.33 – 1.34) | 596.0 ± 80.2 | 12 | 1.33 (1.33 – 1.33) | 625.2 ± 95.8 |
| CB-50 % | 28 | 1.56 (1.56 – 1.56) | 1172.5 ± 172.4 | 15 | 1.56 (1.56 – 1.56) | 1141.1 ± 170.7 | 12 | 1.56 (1.56 – 1.56) | 1200.9 ± 179.0 |
| Cortical bone (1.82 g/cm³) | 22 | 1.82 (1.82 – 1.83) | 1836.4 ± 288.9 | 10 | 1.82 (1.82 – 1.83) | 1791.6 ± 301.8 | 12 | 1.82 (1.82 – 1.82) | 1873.7 ± 285.4 |
| Cortical bone (1.92 g/cm³) | 6 | 1.92 (1.92 – 1.93) | 1922.8 ± 313.0 | 5 | 1.92 (1.92 – 1.93) | 1866.2 ± 313.7 | 0 | 1.92 (1.92 – 1.92) |  |

Table A4. Comparison of CT number for eight tissue-equivalent density plugs used in CT–MD tables registered in Precision, constructed from ClearRT Head FOV 440 mm (Normal) images using full-phantom and half-phantom methods, as well as the combined dataset.

|  |  | Entire data | |  | Full phantom method | |  | Half phantom method | |
| --- | --- | --- | --- | --- | --- | --- | --- | --- | --- |
|  |  | MD (g/cm^3^) | CT number (HU) |  | MD (g/cm^3^) | CT number (HU) |  | MD (g/cm^3^) | CT number (HU) |
| Tissue-equivalent  density plug | n* | Mean (Min-Max) | Mean ± 1SD | n* | Mean (Min-Max) | Mean ± 1SD | n* | Mean (Min-Max) | Mean ± 1SD |
| Air | 21 | 0.001 (0.001 – 0.001) | -1009.0 ± 12.7 | 11 | 0.001 (0.001 – 0.001) | -1012.6 ± 10.3 | 9 | 0.001 (0.001 – 0.001) | -1005.7 ± 15.1 |
| LN-300 Lung | 27 | 0.29 (0.28 – 0.31) | -727.9 ± 41.4 | 15 | 0.29 (0.28 – 0.31) | -732.4 ± 49.0 | 11 | 0.29 (0.29 – 0.30) | -721.2 ± 31.6 |
| LN-450 Lung | 27 | 0.48 (0.45 – 0.50) | -550.7 ± 53.9 | 15 | 0.48 (0.45 – 0.49) | -552.4 ± 61.2 | 11 | 0.49 (0.47 – 0.50) | -548.3 ± 48.0 |
| True water | 26 | 1.00 (1.00 – 1.02) | -71.2 ± 69.5 | 15 | 1.00 (1.00 – 1.02) | -80.5 ± 87.8 | 11 | 1.00 (1.00 – 1.00) | -59.7 ± 38.5 |
| Inner bone | 26 | 1.16 (1.13 – 1.22) | 205.3 ± 108.8 | 15 | 1.16 (1.13 – 1.22) | 209.5 ± 140.4 | 11 | 1.15 (1.13 – 1.21) | 202.9 ± 61.9 |
| CB-30 % | 26 | 1.33 (1.33 – 1.34) | 446.6 ± 100.7 | 14 | 1.33 (1.33 – 1.34) | 439.1 ± 133.2 | 11 | 1.33 (1.33 – 1.33) | 455.6 ± 46.3 |
| CB-50 % | 26 | 1.56 (1.56 – 1.56) | 861.3 ± 135.9 | 14 | 1.56 (1.56 – 1.56) | 843.9 ± 180.8 | 11 | 1.56 (1.56 – 1.56) | 882.6 ± 52.3 |
| Cortical bone (1.82 g/cm³) | 18 | 1.82 (1.82 – 1.83) | 1306.5 ± 235.7 | 14 | 1.82 (1.82 – 1.83) | 1221.2 ± 337.0 | 9 | 1.82 (1.82 – 1.82) | 1377.7 ± 74.0 |
| Cortical bone (1.92 g/cm³) | 8 | 1.92 (1.92 – 1.93) | 1610.5 ± 89.5 | 8 | 1.92 (1.92 – 1.93) | 1629.2 ± 69.4 | 2 | 1.92 (1.92 – 1.92) | 1554.6 ± 153.6 |

Table A5. Comparison of CT number for eight tissue-equivalent density plugs used in CT–MD tables registered in Precision, constructed from ClearRT Thorax FOV 440 mm (Fine) images using full-phantom and half-phantom methods, as well as the combined dataset.

|  |  | Entire data | |  | Full phantom method | |  | Half phantom method | |
| --- | --- | --- | --- | --- | --- | --- | --- | --- | --- |
|  |  | MD (g/cm^3^) | CT number (HU) |  | MD (g/cm^3^) | CT number (HU) |  | MD (g/cm^3^) | CT number (HU) |
| Tissue-equivalent  density plug | n* | Mean (Min-Max) | Mean ± 1SD | n* | Mean (Min-Max) | Mean ± 1SD | n* | Mean (Min-Max) | Mean ± 1SD |
| Air | 26 | 0.001 (0.001 – 0.001) | -1005.2 ± 10.3 | 17 | 0.001 (0.001 – 0.001) | -1006.1 ± 12.7 | 9 | 0.001 (0.001 – 0.001) | -1003.1 ± 1.9 |
| LN-300 Lung | 29 | 0.29 (0.28 – 0.31) | -711.7 ± 16.3 | 19 | 0.29 (0.28 – 0.31) | -708.5 ± 18.5 | 10 | 0.29 (0.28 – 0.30) | -717.7 ± 8.8 |
| LN-450 Lung | 29 | 0.48 (0.45 – 0.50) | -524.4 ± 15.6 | 19 | 0.48 (0.45 – 0.49) | -523.0 ± 17.1 | 10 | 0.49 (0.46 – 0.50) | -526.9 ± 12.6 |
| True water | 29 | 1.00 (1.00 – 1.02) | -15.6 ± 10.7 | 19 | 1.00 (1.00 – 1.02) | -15.9 ± 12.8 | 10 | 1.00 (1.00 – 1.02) | -15.0 ± 5.6 |
| Inner bone | 29 | 1.16 (1.13 – 1.22) | 280.8 ± 54.5 | 19 | 1.16 (1.13 – 1.22) | 278.4 ± 61.2 | 10 | 1.15 (1.13 – 1.21) | 285.3 ± 41.3 |
| CB-30 % | 29 | 1.33 (1.33 – 1.34) | 566.3 ± 81.2 | 19 | 1.33 (1.33 – 1.34) | 546.2 ± 89.9 | 10 | 1.33 (1.33 – 1.33) | 604.5 ± 43.3 |
| CB-50 % | 29 | 1.56 (1.56 – 1.56) | 1089.6 ± 155.6 | 19 | 1.56 (1.56 – 1.56) | 1053.3 ± 172.5 | 10 | 1.56 (1.56 – 1.56) | 1158.5 ± 87.8 |
| Cortical bone (1.82 g/cm³) | 22 | 1.82 (1.82 – 1.83) | 1700.4 ± 232.4 | 13 | 1.82 (1.82 – 1.83) | 1636.3 ± 257.5 | 9 | 1.82 (1.82 – 1.82) | 1793.0 ± 161.1 |
| Cortical bone (1.92 g/cm³) | 7 | 1.92 (1.92 – 1.93) | 1756.6 ± 357.9 | 6 | 1.92 (1.92 – 1.93) | 1712.5 ± 370.6 | 1 | 1.92 (1.92 – 1.92) | 2021.4 † — |

Table A6. Comparison of CT number for eight tissue-equivalent density plugs used in CT–MD tables registered in Precision, constructed from ClearRT Thorax FOV 440 mm (Normal) images using full-phantom and half-phantom methods, as well as the combined dataset.

|  |  | Entire data | |  | Full phantom method | |  | Half phantom method | |
| --- | --- | --- | --- | --- | --- | --- | --- | --- | --- |
|  |  | MD (g/cm^3^) | CT number (HU) |  | MD (g/cm^3^) | CT number (HU) |  | MD (g/cm^3^) | CT number (HU) |
| Tissue-equivalent  density plug | n* | Mean (Min-Max) | Mean ± 1SD | n* | Mean (Min-Max) | Mean ± 1SD | n* | Mean (Min-Max) | Mean ± 1SD |
| Air | 26 | 0.001 (0.001 – 0.001) | -1004.3 ± 11.0 | 26 | 0.001 (0.001 – 0.001) | -1004.3 ± 11.0 | 17 | 0.001 (0.001 – 0.001) | -1005.0 ± 13.5 |
| LN-300 Lung | 29 | 0.29 (0.28 – 0.31) | -679.9 ± 20.4 | 29 | 0.29 (0.28 – 0.31) | -679.9 ± 20.4 | 19 | 0.29 (0.28 – 0.31) | -673.5 ± 22.1 |
| LN-450 Lung | 29 | 0.48 (0.45 – 0.50) | -500.4 ± 21.3 | 29 | 0.48 (0.45 – 0.50) | -500.4 ± 21.3 | 19 | 0.48 (0.45 – 0.50) | -500.2 ± 22.9 |
| True water | 29 | 1.00 (1.00 – 1.02) | -21.4 ± 24.1 | 29 | 1.00 (1.00 – 1.02) | -21.4 ± 24.1 | 19 | 1.00 (1.00 – 1.02) | -23.9 ± 25.8 |
| Inner bone | 29 | 1.16 (1.13 – 1.22) | 238.4 ± 75.2 | 29 | 1.16 (1.13 – 1.22) | 238.4 ± 75.2 | 19 | 1.16 (1.13 – 1.22) | 232.1 ± 88.2 |
| CB-30 % | 29 | 1.33 (1.33 – 1.34) | 508.8 ± 98.6 | 29 | 1.33 (1.33 – 1.34) | 508.8 ± 98.6 | 19 | 1.33 (1.33 – 1.34) | 478.2 ± 98.2 |
| CB-50 % | 29 | 1.56 (1.56 – 1.56) | 990.0 ± 187.5 | 29 | 1.56 (1.56 – 1.56) | 990.0 ± 187.5 | 19 | 1.56 (1.56 – 1.56) | 924.4 ± 193.3 |
| Cortical bone (1.82 g/cm³) | 22 | 1.82 (1.82 – 1.83) | 1512.6 ± 257.0 | 22 | 1.82 (1.82 – 1.83) | 1512.6 ± 257.0 | 13 | 1.82 (1.82 – 1.83) | 1399.0 ± 227.5 |
| Cortical bone (1.92 g/cm³) | 7 | 1.92 (1.92 – 1.93) | 1698.4 ± 334.4 | 7 | 1.92 (1.92 – 1.93) | 1698.4 ± 334.4 | 6 | 1.92 (1.92 – 1.93) | 1660.0 ± 348.9 |

Table A7. Comparison of CT number for eight tissue-equivalent density plugs used in CT–MD tables registered in Precision, constructed from ClearRT Pelvis FOV 440 mm (Fine) images using full-phantom and half-phantom methods, as well as the combined dataset.

|  |  | Entire data | |  | Full phantom method | |  | Half phantom method | |
| --- | --- | --- | --- | --- | --- | --- | --- | --- | --- |
|  |  | MD (g/cm^3^) | CT number (HU) |  | MD (g/cm^3^) | CT number (HU) |  | MD (g/cm^3^) | CT number (HU) |
| Tissue-equivalent  density plug | n* | Mean (Min-Max) | Mean ± 1SD | n* | Mean (Min-Max) | Mean ± 1SD | n* | Mean (Min-Max) | Mean ± 1SD |
| Air | 26 | 0.001 (0.001 – 0.001) | -1005.2 ± 10.3 | 16 | 0.001 (0.001 – 0.001) | -1003.4 ± 5.4 | 9 | 0.001 (0.001 – 0.001) | -1002.0 ± 2.6 |
| LN-300 Lung | 29 | 0.29 (0.28 – 0.31) | -711.7 ± 16.3 | 18 | 0.29 (0.28 – 0.31) | -716.3 ± 11.6 | 10 | 0.29 (0.29 – 0.30) | -717.4 ± 8.0 |
| LN-450 Lung | 29 | 0.48 (0.45 – 0.50) | -524.4 ± 15.6 | 18 | 0.48 (0.45 – 0.49) | -530.5 ± 10.5 | 10 | 0.49 (0.47 – 0.50) | -527.4 ± 11.1 |
| True water | 29 | 1.00 (1.00 – 1.02) | -15.6 ± 10.7 | 18 | 1.00 (1.00 – 1.02) | -13.9 ± 12.8 | 10 | 1.00 (1.00 – 1.00) | -14.0 ± 11.0 |
| Inner bone | 29 | 1.16 (1.13 – 1.22) | 280.8 ± 54.5 | 18 | 1.16 (1.13 – 1.22) | 266.3 ± 67.2 | 10 | 1.15 (1.13 – 1.21) | 257.4 ± 38.2 |
| CB-30 % | 29 | 1.33 (1.33 – 1.34) | 566.3 ± 81.2 | 18 | 1.33 (1.33 – 1.34) | 528.4 ± 61.8 | 10 | 1.33 (1.33 – 1.34) | 558.3 ± 31.5 |
| CB-50 % | 29 | 1.56 (1.56 – 1.56) | 1089.6 ± 155.6 | 18 | 1.56 (1.56 – 1.56) | 991.4 ± 179.8 | 10 | 1.56 (1.56 – 1.56) | 1065.6 ± 68.8 |
| Cortical bone (1.82 g/cm³) | 22 | 1.82 (1.82 – 1.83) | 1700.4 ± 232.4 | 12 | 1.82 (1.82 – 1.83) | 1526.9 ± 229.8 | 9 | 1.82 (1.82 – 1.82) | 1638.8 ± 125.4 |
| Cortical bone (1.92 g/cm³) | 7 | 1.92 (1.92 – 1.93) | 1756.6 ± 357.9 | 6 | 1.92 (1.92 – 1.93) | 1720.0 ± 258.8 | 1 | 1.92 (1.92 – 1.92) | 1888.2 † — |

Table A8. Comparison of CT number for eight tissue-equivalent density plugs used in CT–MD tables registered in Precision, constructed from ClearRT Pelvis FOV 440 mm (Normal) images using full-phantom and half-phantom methods, as well as the combined dataset.

|  |  | Entire data | |  | Full phantom method | |  | Half phantom method | |
| --- | --- | --- | --- | --- | --- | --- | --- | --- | --- |
|  |  | MD (g/cm^3^) | CT number (HU) |  | MD (g/cm^3^) | CT number (HU) |  | MD (g/cm^3^) | CT number (HU) |
| Tissue-equivalent  density plug | n* | Mean (Min-Max) | Mean ± 1SD | n* | Mean (Min-Max) | Mean ± 1SD | n* | Mean (Min-Max) | Mean ± 1SD |
| Air | 26 | 0.001 (0.001 – 0.001) | -1005.2 ± 10.3 | 16 | 0.001 (0.001 – 0.001) | -1005.3 ± 6.7 | 9 | 0.001 (0.001 – 0.001) | -1003.6 ± 3.1 |
| LN-300 Lung | 29 | 0.29 (0.28 – 0.31) | -711.7 ± 16.3 | 18 | 0.29 (0.28 – 0.31) | -675.5 ± 22.8 | 10 | 0.29 (0.29 – 0.30) | -691.1 ± 14.8 |
| LN-450 Lung | 29 | 0.48 (0.45 – 0.50) | -524.4 ± 15.6 | 18 | 0.48 (0.45 – 0.50) | -504.5 ± 22.2 | 10 | 0.49 (0.47 – 0.50) | -516.7 ± 25.3 |
| True water | 29 | 1.00 (1.00 – 1.02) | -15.6 ± 10.7 | 18 | 1.00 (1.00 – 1.02) | -23.1 ± 25.3 | 10 | 1.00 (1.00 – 1.00) | -13.0 ± 18.2 |
| Inner bone | 29 | 1.16 (1.13 – 1.22) | 280.8 ± 54.5 | 18 | 1.16 (1.13 – 1.22) | 222.8 ± 81.7 | 10 | 1.15 (1.13 – 1.21) | 232.5 ± 45.7 |
| CB-30 % | 29 | 1.33 (1.33 – 1.34) | 566.3 ± 81.2 | 17 | 1.33 (1.33 – 1.34) | 455.9 ± 82.0 | 10 | 1.33 (1.33 – 1.34) | 526.3 ± 47.2 |
| CB-50 % | 29 | 1.56 (1.56 – 1.56) | 1089.6 ± 155.6 | 18 | 1.56 (1.56 – 1.56) | 894.9 ± 164.9 | 10 | 1.56 (1.56 – 1.56) | 1023.4 ± 93.3 |
| Cortical bone (1.82 g/cm³) | 22 | 1.82 (1.82 – 1.83) | 1700.4 ± 232.4 | 12 | 1.82 (1.82 – 1.83) | 1324.9 ± 241.2 | 9 | 1.82 (1.82 – 1.82) | 1588.4 ± 204.3 |
| Cortical bone (1.92 g/cm³) | 7 | 1.92 (1.92 – 1.93) | 1756.6 ± 357.9 | 6 | 1.92 (1.92 – 1.93) | 1543.0 ± 364.4 | 1 | 1.92 (1.92 – 1.92) | 1666.9 † — |

Table A9. Comparison of CT number for eight tissue-equivalent density plugs used in CT–MD tables registered in Precision, constructed from ClearRT Whole Body FOV 270 mm (Fine) images using full-phantom and half-phantom methods, as well as the combined dataset.

|  |  | Entire data | |  | Full phantom method | |  | Half phantom method | |
| --- | --- | --- | --- | --- | --- | --- | --- | --- | --- |
|  |  | MD (g/cm^3^) | CT number (HU) |  | MD (g/cm^3^) | CT number (HU) |  | MD (g/cm^3^) | CT number (HU) |
| Tissue-equivalent  density plug | n* | Mean (Min-Max) | Mean ± 1SD | n* | Mean (Min-Max) | Mean ± 1SD | n* | Mean (Min-Max) | Mean ± 1SD |
| Air | 20 | 0.001 (0.001 – 0.001) | -1012.9 ± 14.3 | 12 | 0.001 (0.001 – 0.001) | -1016.5 ± 14.9 | 8 | 0.001 (0.001 – 0.001) | -1009.2 ± 12.6 |
| LN-300 Lung | 27 | 0.29 (0.28 – 0.31) | -754.3.1 ± 62.8 | 17 | 0.29 (0.28 – 0.31) | -765.5 ± 73.8 | 12 | 0.29 (0.28 – 0.30) | -735.3 ± 30.0 |
| LN-450 Lung | 27 | 0.48 (0.45 – 0.50) | -570.7 ± 59.2 | 17 | 0.48 (0.45 – 0.50) | -579.6 ± 67.7 | 12 | 0.49 (0.47 – 0.50) | -554.3 ± 36.1 |
| True water | 27 | 1.00 (1.00 – 1.02) | -61.6 ± 59.4 | 17 | 1.00 (1.00 – 1.02) | -70.2 ± 67.3 | 12 | 1.00 (1.00 – 1.00) | -47.9 ± 38.1 |
| Inner bone | 26 | 1.15 (1.13 – 1.22) | 191.0 ± 104.3 | 17 | 1.16 (1.13 – 1.22) | 181.5 ± 122.2 | 11 | 1.15 (1.13 – 1.21) | 199.2 ± 58.1 |
| CB-30 % | 26 | 1.33 (1.27 – 1.34) | 436.3 ± 80.4 | 16 | 1.33 (1.27 – 1.34) | 417.1 ± 89.8 | 12 | 1.33 (1.33 – 1.33) | 463.8 ± 48.9 |
| CB-50 % | 25 | 1.56 (1.56 – 1.56) | 856.3 ± 107.1 | 16 | 1.56 (1.56 – 1.56) | 828.0 ± 112.3 | 11 | 1.56 (1.56 – 1.56) | 891.9 ± 78.7 |
| Cortical bone (1.82 g/cm³) | 17 | 1.82 (1.82 – 1.83) | 1315.3 ± 149.2 | 10 | 1.82 (1.82 – 1.83) | 1248.2 ± 131.9 | 9 | 1.82 (1.82 – 1.82) | 1381.6 ± 123.5 |
| Cortical bone (1.92 g/cm³) | 8 | 1.92 (1.92 – 1.93) | 1548.3 ± 92.3 | 6 | 1.92 (1.92 – 1.93) | 1561.2 ± 85.6 | 2 | 1.92 (1.92 – 1.92) | 1509.5 ± 137.9 |

Table A10. Comparison of CT number for eight tissue-equivalent density plugs used in CT–MD tables registered in Precision, constructed from ClearRT Whole Body FOV 270 mm (Normal) images using full-phantom and half-phantom methods, as well as the combined dataset.

|  |  | Entire data | |  | Full phantom method | |  | Half phantom method | |
| --- | --- | --- | --- | --- | --- | --- | --- | --- | --- |
|  |  | MD (g/cm^3^) | CT number (HU) |  | MD (g/cm^3^) | CT number (HU) |  | MD (g/cm^3^) | CT number (HU) |
| Tissue-equivalent  density plug | n* | Mean (Min-Max) | Mean ± 1SD | n* | Mean (Min-Max) | Mean ± 1SD | n* | Mean (Min-Max) | Mean ± 1SD |
| Air | 22 | 0.001 (0.001 – 0.001) | -1010.7 ± 12.4 | 16 | 0.001 (0.001 – 0.001) | -1017.1 ± 14.4 | 10 | 0.001 (0.001 – 0.001) | -1009.3 ± 13.6 |
| LN-300 Lung | 27 | 0.29 (0.28 – 0.31) | -731.3 ± 40.9 | 18 | 0.29 (0.28 – 0.31) | -765.5 ± 71.6 | 12 | 0.29 (0.28 – 0.30) | -719.5 ± 19.6 |
| LN-450 Lung | 27 | 0.48 (0.45 – 0.50) | -552.8 ± 52.8 | 18 | 0.48 (0.45 – 0.50) | -578.5 ± 65.8 | 12 | 0.49 (0.46 – 0.50) | -541.3 ± 27.9 |
| True water | 27 | 1.00 (1.00 – 1.02) | -67.7 ± 80.8 | 18 | 1.00 (1.00 – 1.02) | -68.4 ± 65.7 | 12 | 1.00 (1.00 – 1.00) | -59.2 ± 39.6 |
| Inner bone | 25 | 1.15 (1.13 – 1.22) | 167.1 ± 127.3 | 18 | 1.16 (1.13 – 1.22) | 181.5 ± 122.2 | 11 | 1.15 (1.13 – 1.21) | 173.1 ± 60.6 |
| CB-30 % | 26 | 1.33 (1.27 – 1.34) | 398.0 ± 125.0 | 17 | 1.33 (1.27 – 1.34) | 418.9 ± 87.3 | 12 | 1.33 (1.27 – 1.33) | 439.3 ± 67.6 |
| CB-50 % | 25 | 1.56 (1.56 – 1.56) | 789.2 ± 176.9 | 18 | 1.56 (1.56 – 1.56) | 828.0 ± 112.3 | 11 | 1.56 (1.56 – 1.56) | 847.7 ± 114.5 |
| Cortical bone (1.82 g/cm³) | 17 | 1.82 (1.82 – 1.83) | 1230.0 ± 202.4 | 12 | 1.82 (1.82 – 1.83) | 1248.2 ± 131.9 | 10 | 1.82 (1.82 – 1.82) | 1305.2 ± 163.7 |
| Cortical bone (1.92 g/cm³) | 7 | 1.92 (1.92 – 1.93) | 1494.9 ± 65.5 | 6 | 1.92 (1.92 – 1.93) | 1561.2 ± 85.6 | 1 | 1.92 (1.92 – 1.92) | 1546.3 † — |

Table S1. Comparison of CT–MD Calibration Data for the Overall Data Set and the Matched‑Conditions Subgroup

|  |  | Entire data | |  | Subgroup data | |
| --- | --- | --- | --- | --- | --- | --- |
|  |  | MD (g/cm^3^) | CT number (HU) |  | MD (g/cm^3^) | CT number (HU) |
| Tissue-equivalent |  | Mean (Min-Max) | Mean ± 1SD | n* | Mean (Min-Max) | Mean ± 1SD |
| density plug | n* |  |  |  |  |  |
| Air | 34 | 0.001 (0.001 – 0.01) | -995.9 ± 12.9 | 9 | 0.001 (0.001 – 0.01) | -989.4 ± 17.7 |
| LN-300 Lung | 32 | 0.29 (0.26 – 0.30) | -703.6 ± 14.4 | 8 | 0.29 (0.29 – 0.30) | -694.7 ± 12.9 |
| LN-450 Lung | 34 | 0.48 (0.45 – 0.50) | -518.8 ± 17.5 | 9 | 0.48 (0.45 – 0.50) | -505.5 ± 8.6 |
| True water | 34 | 1.00 (1.00 – 1.00) | 1.4 ± 6.3 | 9 | 1.00 (1.00 – 1.00) | 4.7 ± 4.8 |
| Inner bone | 32 | 1.14 (1.11 – 1.16) | 219.9 ± 11.2 | 9 | 1.14 (1.13 – 1.16) | 222.0 ± 9.8 |
| CB-30 % | 31 | 1.33 (1.33 – 1.34) | 468.3 ± 28.1 | 8 | 1.33 (1.33 – 1.34) | 469.3 ± 10.0 |
| CB-50 % | 34 | 1.56 (1.53 – 1.56) | 848.0 ± 48.8 | 9 | 1.56 (1.53 – 1.56) | 854.1 ± 42.3 |
| Cortical bone (1.82 g/cm³) | 32 | 1.82 (1.82 – 1.83) | 1284.5 ± 78.5 | 9 | 1.82 (1.82 – 1.83) | 1289.6 ± 78.2 |

Table S1 compares the entire data (n = 31–34 per plug) with the matched‑conditions subgroup data (FOV = 500 mm & Sun Nuclear Solid Water Tomo phantom; n = 8–9 per plug).
